# Longitudinal associations of DNA methylation and sleep in children: a meta-analysis

**DOI:** 10.1186/s13148-022-01298-4

**Published:** 2022-07-05

**Authors:** Sara Sammallahti, M. Elisabeth Koopman-Verhoeff, Anne-Claire Binter, Rosa H. Mulder, Alba Cabré-Riera, Tuomas Kvist, Anni L. K. Malmberg, Giancarlo Pesce, Sabine Plancoulaine, Jonathan A. Heiss, Sheryl L. Rifas-Shiman, Stefan W. Röder, Anne P. Starling, Rory Wilson, Kathrin Guerlich, Kristine L. Haftorn, Christian M. Page, Annemarie I. Luik, Henning Tiemeier, Janine F. Felix, Katri Raikkonen, Jari Lahti, Caroline L. Relton, Gemma C. Sharp, Melanie Waldenberger, Veit Grote, Barbara Heude, Isabella Annesi-Maesano, Marie-France Hivert, Ana C. Zenclussen, Gunda Herberth, Dana Dabelea, Regina Grazuleviciene, Marina Vafeiadi, Siri E. Håberg, Stephanie J. London, Mònica Guxens, Rebecca C. Richmond, Charlotte A. M. Cecil

**Affiliations:** 1 Department of Adolescent and Child Psychiatry, Erasmus MC, University Medical Center Rotterdam, Rotterdam, The Netherlands; 2 Department of Obstetrics and Gynaecology, University of Helsinki and Helsinki University Hospital, Helsinki, Finland; 3 Generation R Study Group, Erasmus MC, University Medical Center Rotterdam, Rotterdam, The Netherlands; 4 Institute of Education and Child Studies, Leiden University, Leiden, The Netherlands; 5 Barcelona Institute for Global Health, ISGlobal, Campus Mar, Doctor Aiguader, 88, 08003 Barcelona, Spain; 6 Universitat Pompeu Fabra, Barcelona, Spain; 7 Spanish Consortium for Research on Epidemiology and Public Health (CIBERESP), Instituto de Salud Carlos III, Madrid, Spain; 8 Department of Psychology and Logopedics, University of Helsinki, Helsinki, Finland; 9 INSERM UMR-S 1136, Team of Epidemiology of Allergic and Respiratory Diseases (EPAR), Institute Pierre Louis of Epidemiology and Public Health (IPLESP), Sorbonne University, Paris, France; 10 CRESS, Inserm, INRAE, Université de Paris Cité, Paris, France; 11 Department of Environmental Medicine and Public Health, Icahn School of Medicine at Mount Sinai, New York, NY USA; 12 Department of Population Medicine, Harvard Medical School, Harvard Pilgrim Health Care Institute, Boston, MA USA; 13 Department of Environmental Immunology, Helmholtz Centre for Environmental Research - UFZ, Leipzig, Germany; 14 Department of Epidemiology, Colorado School of Public Health, University of Colorado Anschutz Medical Campus, Aurora, CO USA; 15 Center for Lifecourse Epidemiology of Adiposity and Diabetes, University of Colorado Anschutz Medical Campus, Aurora, CO USA; 16 Department of Epidemiology, University of North Carolina at Chapel Hill, Chapel Hill, NC USA; 17 Research Unit Molecular Epidemiology, Institute of Epidemiology, Helmholtz Zentrum München, German Research Center for Environmental Health, Neuherberg, Bavaria Germany; 18 Division of Metabolic and Nutritional Medicine, Department of Pediatrics, Dr. Von Hauner Children’s Hospital, LMU University Hospital Munich, Munich, Germany; 19 Department of Genetics and Bioinformatics, Norwegian Institute of Public Health, Oslo, Norway; 20 Centre for Fertility and Health, Norwegian Institute of Public Health, Oslo, Norway; 21 Institute of Health and Society, University of Oslo, Oslo, Norway; 22 Department of Mathematics, University of Oslo, Oslo, Norway; 23 Department of Epidemiology, Erasmus MC, University Medical Center Rotterdam, Rotterdam, The Netherlands; 24 Department of Social and Behavioral Science, Harvard T.H. Chan School of Public Health, Boston, MA USA; 25 Department of Pediatrics, Erasmus MC, University Medical Center Rotterdam, Rotterdam, The Netherlands; 26 MRC Integrative Epidemiology Unit, University of Bristol, Bristol, UK; 27 Population Health Sciences, Bristol Medical School, University of Bristol, Bristol, UK; 28 IDESP, University of Montpellier and INSERM, Montpellier, France; 29 Perinatal Immunology Group, Saxonian Incubator for Clinical Translation - SIKT, Leipzig University, Leipzig, Germany; 30 Department of Pediatrics, University of Colorado School of Medicine, University of Colorado Anschutz Medical Campus, Aurora, CO USA; 31 Department of Environmental Science, Vytautas Magnus University, Kaunas, Lithuania; 32 Department of Social Medicine, Faculty of Medicine, University of Crete, Heraklion, Crete Greece; 33 Epidemiology Branch, Department of Health and Human Services, National Institute of Environmental Health Sciences, National Institutes of Health, Research Triangle Park, NC USA; 34 Molecular Epidemiology, Department of Biomedical Data Sciences, Leiden University Medical Center, Leiden, The Netherlands; 35 Department of Psychology, Institute of Psychology, Psychiatry and Neuroscience, King’s College London, London, UK

**Keywords:** Sleep, Methylation, Epigenomics, Actigraphy, Child, Meta-analysis, Longitudinal studies

## Abstract

**Background:**

Sleep is important for healthy functioning in children. Numerous genetic and environmental factors, from conception onwards, may influence this phenotype. Epigenetic mechanisms such as DNA methylation have been proposed to underlie variation in sleep or may be an early-life marker of sleep disturbances. We examined if DNA methylation at birth or in school age is associated with parent-reported and actigraphy-estimated sleep outcomes in children.

**Methods:**

We meta-analysed epigenome-wide association study results. DNA methylation was measured from cord blood at birth in 11 cohorts and from peripheral blood in children (4–13 years) in 8 cohorts. Outcomes included parent-reported sleep duration, sleep initiation and fragmentation problems, and actigraphy-estimated sleep duration, sleep onset latency and wake-after-sleep-onset duration.

**Results:**

We found no associations between DNA methylation at birth and parent-reported sleep duration (*n* = 3658), initiation problems (*n* = 2504), or fragmentation (*n* = 1681) (*p* values above cut-off 4.0 × 10^–8^). Lower methylation at *cg24815001* and *cg02753354* at birth was associated with longer actigraphy-estimated sleep duration (*p* = 3.31 × 10^–8^, *n* = 577) and sleep onset latency (*p* = 8.8 × 10^–9^, *n* = 580), respectively. DNA methylation in childhood was not cross-sectionally associated with any sleep outcomes (*n* = 716–2539).

**Conclusion:**

DNA methylation, at birth or in childhood, was not associated with parent-reported sleep. Associations observed with objectively measured sleep outcomes could be studied further if additional data sets become available.

**Supplementary Information:**

The online version contains supplementary material available at 10.1186/s13148-022-01298-4.

## Introduction

Sleep is a key aspect of healthy functioning; disruptions in sleep as early as during childhood have been linked to a wide range of mental and physical health problems [1]. Sleep is a complex, multifactorial phenotype, reflecting the influence of both genetic and environmental factors, beginning in utero. For example, prenatal exposure to maternal smoking [2], alcohol use [3] and depression [4, 5] during pregnancy have been linked to disrupted sleep in children. However, the biological factors underlying sleep development in childhood remain unclear.

Epigenetic processes have received much attention as a biological mechanism that may underlie differences in child development and mediate the effects of foetal or early-life exposures on later health. The most widely studied of epigenetic processes, DNA methylation (DNAm) of cytosine nucleotides followed by guanine (CpGs), has been linked to a variety of mental and physical health outcomes [6, 7], and to foetal exposures such as maternal smoking [8]. It has been proposed that DNAm may also serve as a mechanism by which genetic and environmental exposures influence sleep [9]. DNAm is also showing promise as a biological marker for disease prediction, early detection and risk stratification. This application is especially well suited for use in peripheral tissues, which are more readily available in humans but may not be causal for the phenotype of interest. For example, it is already possible to estimate a range of exposures, traits and health outcomes based on peripheral DNAm patterns alone (e.g. age, smoking, BMI) [10].

While associations between epigenetic changes and sleep have been previously assessed, these have mainly been investigated within the context of experimental studies of sleep deprivation or sleep disruption experienced during shift work [11–13]. Studies on DNAm in relation to sleep characteristics among children or adolescents are scarce. One epigenome-wide study utilizing a network-based approach identified a module of inter-correlated CpG sites in peripheral blood associated with actigraphy-estimated sleep duration among 10-year-olds (*n* = 188) [14]. Another epigenome-wide pilot study reported an association between blood DNAm and diary-based sleep duration among 18- to 19-year-olds (*n* = 26) [15], and a third epigenome-wide study reported differences in buccal cell DNAm between 18- and 19-year-old monozygotic twins who were discordant for diurnal preference (*n* = 30) [16]. One candidate region study reported an association between DNAm of long interspersed nuclear elements and metabolism-related genes in leukocytes, and actigraphy-estimated sleep duration and fragmentation among 14-year-olds (*n* = 351) [17]. These prior studies, while encouraging as proof-of-concept, are limited due to small sample size and cross-sectional design, and vulnerable to chance findings due to single-cohort design.

Since in utero exposures and genetic factors are known to influence sleep outcomes later in life, we posit that DNAm at birth may be an early-life marker of sleep disturbances [18]. Our primary aim was to investigate if DNAm (at birth) is prospectively associated with parent-reported sleep duration in children. As secondary aims, we investigated two other parent-reported sleep-related phenotypes (sleep fragmentation or initiation problems) and three actigraphy-estimated sleep measures (duration, sleep onset latency and wake-after-sleep-onset duration). In further analyses, we also investigated associations between childhood (4–13 years) DNAm and sleep characteristics. For the current meta-analysis, a total of 14 cohorts in the Pregnancy And Childhood Epigenetics (PACE) consortium conducted one or multiple epigenome-wide association study (EWAS) analyses to address the primary and secondary aims of our study (*n* = 3658 participants in primary analysis).

## Methods

### Participating studies

Fourteen North American and European cohorts, all members of the Pregnancy and Childhood Epigenetics Consortium (PACE) [19], participated in this meta-analysis. Eleven cohorts had data on newborn cord blood DNAm at birth and child sleep (Table 1): of these, ten cohorts had data on parent-reported sleep outcomes and three had data on actigraphy-estimated sleep outcomes. Eight cohorts had data on DNAm in peripheral blood in childhood and child sleep (Additional file 1: Table S1). Overlap within each cohort was large across data sets, i.e. same individuals formed the majority of the analytical sample in different cohort-level analyses of, for example, DNAm at birth and DNAm in childhood (within the same cohort): in total, five cohorts had data on DNAm both in cord blood and in peripheral blood in childhood (Additional file 1: Table S2). Individual cohorts are described in more detail in Additional file 2: Methods.

**Table 1:** Characteristics of the participating cohorts in analyses of cord blood DNAm and child sleep outcomes

	ALSPAC	EDEN	Generation R	Healthy Start	INMA	LINA	MoBa-1	MoBa-2	PREDO	PROGRESS	Viva
Country	UK	France	Netherlands	USA	Spain	Germany	Norway	Norway	Finland	Mexico	USA
Children with DNAm at birth and sleep data, n	855	122	597	283	260	204	711	415	247	244	410
DNAm array type for cord blood	450 K	450 K	450 K	450 K	450 K	450 K	450 K	450 K	450 K	EPIC	450 K
*Parent-reported child sleep*											
Number of children with data, n (%)	755 ^a^	122	252 ^b^	283	260	203 ^c^	711	415	247	0	410
Child age at assessment, years, mean (SD)	11.7 (0.1)	5.7 (0.1)	11.7 (0.2)	4.7 (0.6)	11.1 (0.5)	10.1 (0.3)	7.0 (0.2)	7.1 (0.3)	3.9 (0.7)	n/a	7.8 (0.7)
Duration, hours, mean (SD)	9.9 (0.6)	10.8 (0.5)	9.5 (0.7)	9.4 (0.9)	9.5 (0.5)	9.5 (0.9)	10.2 (0.7)	10.1 (0.7)	10.2 (0.7)	n/a	9.8 (0.9)
Initiation difficulties, yes, n (%)	434 (53.1)	46 (37.4)	150 (23.4)	87 (30.7)	222 (85.4)	34 (16.7)	n/a	n/a	82 (33.2)	n/a	n/a
Sleep fragmentation, yes, n (%)	86 (10.8)	43 (35.0)	n/a	32 (11.3)	42 (16.2)	n/a	n/a	n/a	87 (35.2)	n/a	n/a
*Actigraphy-estimated child sleep*											
Number of children with data, n (%)	0	0	257	0	81	0	0	0	0	244	0
Child age at assessment, years, mean (SD)	n/a	n/a	11.7 (0.2)	n/a	11.1 (0.5)	n/a	n/a	n/a	n/a	4.7 (0.5)	n/a
Duration (total sleep time), hours, mean (SD)	n/a	n/a	7.6 (0.7)	n/a	7.2 (1.0)	n/a	n/a	n/a	n/a	8.0 (0.6)	n/a
Sleep-onset latency, minutes, mean (SD)	n/a	n/a	42 (46.8)	n/a	6.8 (15.4)	n/a	n/a	n/a	n/a	10.6 (10.6)	n/a
Wake-after-sleep-onset duration, minutes, mean (SD)	n/a	n/a	86.0 (33.0)	n/a	41.2 (23.3)	n/a	n/a	n/a	n/a	102.8 (33.0)	n/a
*Maternal characteristics*											
Education, low, n (%) ^d^	417 (49.8)	29 (23.6)	48 (7.5)	92 (32.5)	63 (24.2)	4 (2.0)	258 (36.3)	157 (37.8)	13 (5.3)	103 (42.2)	6 (1.5)
Age, years, mean (SD)	30.1 (4.4)	30.3 (5.0)	31.9 (3.8)	27.3 (6.1)	30.6 (4.0)	30.8 (4.6)	29.9 (4.3)	30.3 (4.4)	33.5 (5.8)	28.2 (5.4)	32.3 (5.2)
Smoking during pregnancy, n (%)											
No smoking during pregnancy	727 (87.2)	94 (76.4)	506 (79.1)	241 (85.2)	187 (71.9)	201 (98.5)	521 (73.7)	316 (76.1)	234 (94.7)	n/a	374 (91.2)
Smoked during pregnancy	107 (12.8)	29 (23.6)	134 (20.9)	42 (14.8)	73 (28.1)	3 (1.5)	190 (26.7)	99 (23.9)	13 (5.3)	n/a	36 (8.8)
Quit in early pregnancy	27 (3.2)	9 (7.3)	61 (9.5)	20 (7.1)	40 (15.4)	n/a	99 (13.9)	62 (14.9)	9 (3.6)	n/a	n/a
Continued smoking	80 (9.6)	20 (16.3)	73 (11.4)	22 (7.8)	33 (12.7)	n/a	91 (12.8)	37 (8.9)	4 (1.6)	n/a	n/a
*Child characteristics*											
Sex, female, n (%)	441 (51.6)	52 (42.3)	319 (49.8)	134 (47.3)	123 (47.3)	97 (47.5)	328 (46.1)	188 (45.3)	117 (47.4)	112 (45.9)	199 (48.5)
Gestational age, weeks, mean (SD)	39.5 (1.5)	39.4 (1.5)	40.2 (1.4)	39.4 (1.2)	39.8 (1.3)	39.8 (1.4)	39.6 (1.5)	39.4 (1.6)	39.7 (3.1)	38.4 (1.6)	39.7 (1.6)

*450 K* Illumina Infinium^®^ HumanMethylation450 BeadChip; *DNAm* deoxyribonucleic acid methylation; *EPIC* Illumina Infinium^®^ HumanMethylationEPIC BeadChip; *kg* kilogram; *n* number of participants; *n/a* not applicable due to lack of available data; *SD* standard deviation; *UK* United Kingdom; *USA* United States of America

^a^In ALSPAC, sample size and age varied per outcome: of those with DNAm data at birth, 755 children had data on parent-reported sleep duration at the mean age of 11.7 years (SD = 0.1), while 791 and 769 children had data on parent-reported sleep initiation and fragmentation problems at the age of 9.6 years (SD = 0.1), respectively

^b^In Generation R, sample size and age varied per outcome: of those with DNAm data at birth, 252 children had data on parent-reported sleep duration at the mean age of 11.7 years (SD = 0.2), while 597 children had data on parent-reported sleep initiation problems at the mean age of 9.7 years (SD = 0.3)

^c^In LINA, 203 children had data on parent-reported sleep duration and 204 children had data on parent-reported sleep initiation problems

^d^Rates of low education are not directly comparable, as educational systems differed between countries and cohorts used different definitions of low education, as explained in more detail in Additional file 2: Methods

### Measures

#### Exposure: DNA methylation at birth and in childhood

Cohorts collected samples of newborn blood from umbilical cord blood at birth (Table 1) and child blood using venepuncture at 4–13 years of age (Additional file 1: Table S1). DNAm was assessed with the *Illumina*^®^* HumanMethylation450* (450 k) or the *HumanMethylationEPIC* (EPIC) *BeadChip* assay at Illumina or cohort-specific laboratories. Cohorts performed sample processing, quality control and normalization as described in Additional file 2: Methods.

We used normalized, untransformed DNAm beta values, ranging from 0 (completely unmethylated) to 1 (completely methylated), after trimming extreme outliers (3 × interquartile range from the quartile limit). Certain individuals were removed due to trimming of extreme values, resulting in different sample sizes across probes and cohorts. We excluded probes mapped to X or Y chromosomes, polymorphic CpGs which overlap with known single-nucleotide polymorphisms, probes with cohort-level call rate < 90%, control probes and cross-reactive probes (targeting repetitive sequences/co-hybridizing to alternate sequences) [20, 21].

#### Outcomes: parent-reported and actigraphy-estimated sleep measures

*Parent-reported sleep duration* was chosen as the primary outcome because of known associations with genetic and early-life environmental factors, comparability across studies, and large sample size (*n* = 3658, for other phenotypes *n* ≤ 2504) [1]. Sleep duration was the parent-reported number of hours the child slept per night (e.g. *"How many hours of sleep does your child get on most nights?")* or the calculated difference between reported average time of falling asleep and waking up. This primary outcome was standardized within each cohort (mean = 0, SD = 1).

We also included parent-reported sleep initiation problems (e.g. *"The child has difficulty getting to sleep at night"*) and sleep fragmentation problems (e.g. "*The child wakes up more than twice per night*") to consider multiple dimensions of sleep [22, 23]. These were used as secondary, binary outcomes.

*Actigraphy-estimated* sleep outcomes were chosen to objectively assess determined sleep characteristics. Sleep duration (total sleep time), sleep onset latency (time between lying down in bed and falling asleep) and wake-after-sleep-onset duration (time awake between falling asleep and final awakening) were recorded using accelerometery and averaged across the measurement period of 3–9 days. These outcomes were all standardized within each sample (mean = 0, SD = 1). Four cohorts used wrist-worn actigraphs to measure sleep (*GENEActiv* in Generation R and INMA; *Actigraph GT3X* in PROGRESS; *Actiwatch AW7* in GLAKU). One cohort (CHOP) used armband accelerometers (*SenseWear Armband*): this cohort only had childhood DNAm, not newborn DNAm data, and thus only contributed to sensitivity analyses (see “*Statistical analyses*”).

Mean child age at parental assessment ranged between 4 and 12 years between studies. Mean age at actigraphy was 11–12 years in four cohorts and 4.7 years in one cohort (PROGRESS). Please see Table 1 and Additional file 1: Table S1 for cohort-level descriptives and Additional file 2: Methods for further details.

#### Covariates

We adjusted for maternal smoking, maternal age, maternal education, child sex, child age at sleep assessment, cell counts, and surrogate variables for batch adjustment. Maternal smoking and education were coded and categorized according to data availability as explained in Additional file 2: Methods. Cell counts were estimated using the Houseman method [24] with the Bakulski reference panel [25] (cord blood samples) and Reinius reference panel [26] (childhood samples). In cord blood DNAm analyses, we additionally adjusted for gestational age at birth, and in childhood DNAm analyses, we adjusted for child age at venepuncture, if different from age at sleep assessment (Table S1). Covariates were chosen based on the previous literature to increase precision and to address potential confounders such as maternal smoking and socio-economic status [2, 8, 27, 28]. Cohort-level analysts were advised to adjust for ethnic differences in any multi-ethnic cohort by choosing the appropriate approach based on cohort characteristics and data availability: these cohort-specific covariates were country of birth (CHOP), self-reported ethnicity (Healthy Start, Viva), or principal components from genome-wide sequencing data (GLAKU, PREDO) (Additional file 2: Methods).

### Statistical analyses

#### Cohort-level EWAS

Each cohort-level EWAS was performed according to a predefined analysis plan. We used multivariate linear and logistic regression, for continuous (sleep duration and actigraphy-estimated outcomes) and binary (parent-reported sleep initiation and fragmentation) outcomes, respectively. All *p* values were two-sided. We excluded participants with missing data and siblings (one sibling per sibling pair, chosen at random). For more cohort-level information, please see Additional file 2: Methods.

#### Primary meta-analysis: newborn DNAm and parent-reported sleep duration

We examined associations between newborn cord blood DNAm and parent-reported sleep duration at 364,672 loci across 10 cohorts (max *n* = 3658). We combined 450 k and EPIC data, only including sites that are available on the 450 k array [29]. We performed an inverse variance-weighted fixed-effects meta-analysis using R-3.6.1 (https://www.r-project.org) and METAL (release 2018–08-28) [30]. To assess epigenome-wide statistical inflation, we calculated cohort- and meta-analysis-level genomic inflation factor lambdas (λ) and examined quantile–quantile plots (see *Results*).

Cohort-level results were meta-analysed at Erasmus MC. We verified the findings in shadow meta-analyses conducted independently at ISGlobal, using the meta-analytical tool GWAMA instead of METAL [31].

#### Sensitivity analyses

We reran the primary meta-analysis when including only (1) cohorts based in Europe, and (2) cohorts where sleep was assessed on school-aged children (i.e. mean age ≥ 7 years, corresponding to the highest primary school entrance age in the studied populations, http://uis.unesco.org/sites/default/files/documents/indicator-efa-official-entrance-age-to-primary-education.xlsx, accessed 2021/06/09), to increase precision at the cost of sample size.

Individual variations in cord and peripheral blood DNAm are only partly stable from birth to school age [32]. To check if the timing of the DNAm measurement changed the findings, cohorts with available data (re-)ran the EWAS using DNAm data from blood samples collected at school age, and we then reran the meta-analysis on these cohort-level results.

As a final sensitivity analysis, we repeated the primary meta-analysis using a sample-size-weighted meta-analytical approach (also in METAL), which does not expect effect magnitude to be similar across cohorts, to complement the primary inverse variance-weighted approach.

U-shaped or other nonlinear associations were not tested for lack of a strong hypothesis.

#### Meta-analyses of secondary phenotypes

We ran five secondary meta-analyses that were otherwise similar to the primary meta-analysis but utilized alternative outcomes: parent-reported (1) sleep initiation and (2) fragmentation problems, and actigraphy-estimated (3) sleep duration, (4) sleep-onset latency, and (5) wake-after-sleep-onset duration.

#### Multiple testing correction and subthreshold findings

The DNAm of nearby sites is correlated, and a cut-off of 2.4 × 10^–7^ for multiple testing correction on 450 k-based EWASes has been recommended [33]. In the current study, we conducted one primary and five secondary meta-analyses on six separate outcomes: the cut-off of epigenome-level hit was thus (2.4 × 10^–7^)/6 = 4.0 × 10^–8^. To confirm independence among the outcomes, we extracted eigenvalues from individual-level matrix of phenotype data using the *meff* function from *poolr* (Additional file 1: Figure S1).

Statistical significance cut-offs are inescapably somewhat arbitrary, and particularly in the case of borderline-significant findings, more information on effect magnitude and consistency across studies may be needed to balance type I and II error. We used a suggestive cut-off of *p* < 5.0 × 10^−5^ to select the top 25 subthreshold findings that came closest to statistical significance in our primary meta-analysis: this cut-off was selected in line with a recent meta-analysis on maternal anxiety and DNAm at birth [34]. In supplementary tables, we report the site-level results of the primary meta-analysis and sensitivity analyses (as described above) for these sites.

#### Annotation and description of CpGs

Probes were annotated using *meffil* (hg19/b37) [35]. Previously reported EWAS-based associations between CpGs of interest and child/adult phenotypes were derived from the EWAS catalogue (http://ewascatalog.org/ accessed 2021/06/09) [36]. Function-related information was derived from Gene-Cards (https://www.genecards.org accessed 2021/06/09) and GWAS Catalog (https://www.ebi.ac.uk/gwas accessed 2021/06/09). For look-up analyses, single-nucleotide polymorphisms related to sleep duration in previous genome-wide-association studies [37–41] were identified using the Sleep Disorder Knowledge Portal (https://sleep.hugeamp.org, accessed 2021/06/23).

We then follow up the hits (none in the primary meta-analysis, two hits in the secondary meta-analyses) to report cross-tissue correspondence at these loci between DNAm in blood vs in the brain, the most relevant organ for sleep phenotype. We used previously published data on DNAm correlations across blood and brain tissue (http://epigenetics.essex.ac.uk/bloodbrain/ accessed 2021/06/09) [42] and circadian expression of genes of interest in human and mouse tissues (http://circadb.hogeneschlab.org/human, accessed 2021/06/21).

#### Gene ontology

To test for functional enrichment of annotated genes, we performed gene ontology (GO) pathway analyses on genes annotated to CpGs with the lowest p values (*p* < 1.0 × 10^–4^) for each model separately (1 primary + 5 secondary meta-analyses), using *missMethyl* in R [43]. The number of CpGs in these analyses was 44, 62, 51, 60, 43 and 85, for analyses on DNAm at birth and parent-rated sleep duration (primary meta-analysis), initiation problems, fragmentation, actigraphy-estimated sleep duration, sleep onset latency and wake-after-sleep-onset duration, respectively. Analyses were adjusted for the number of pathways tested based on the false discovery rate (FDR) [44].

#### DMR analyses

We used the output of the primary and secondary meta-analyses to identify DMRs in newborn cord blood associated with parent-reported child sleep duration, initiation and fragmentation, actigraphy-estimated sleep duration, sleep-onset latency and wake-after-sleep-onset duration.

DMRs were analysed using two alternative approaches: *DMRcate* [45] and *ipDMR* [46]. Briefly, *DMRcate* applies Gaussian kernel smoothing for t-statistics using a bandwidth lambda, and p values for regions are calculated based on the Satterthwaite method and corrected with FDR [45]. On the other hand, *ipDMR* calculates p values for intervals bordered by two adjacent CpGs, performs the Benjamini–Hochberg (BH) procedure on the interval p values to select those significant intervals at a user-specified FDR threshold (seed threshold), joins all nearby significant intervals and CpGs, recalculates *p* values for each combined region using the original p values for all CpGs and then obtains FDR-adjusted p values for these regions [46]. In *DMRcate*, alpha level for FDR as calculated on individual CpGs was 0.05; in ipDMR, FDR alpha level for initial selection of regions (seed) was also set at 0.05 [46], *DMRcate* lambda = 500 and C = 5 were set as previously recommended [47], otherwise default parameters for both approaches were applied.

## Results

### Study characteristics

Associations between newborn DNAm and child sleep were assessed in 11 cohorts, whose participants are described in Table 1. Of these 11 cohorts, 10 had data on parent-reported sleep outcomes and 3 had data on actigraphy-estimated outcomes. Two of these cohorts (Generation R, INMA) had both parent-reported and actigraphy-estimated sleep data: correlations across all different sleep outcomes within these cohorts are shown in Additional file 1: Figure S1.

Associations between *childhood* DNAm and child sleep were assessed in 8 cohorts, described in Additional file 1: Table S1. Five of these cohorts had DNAm data available among both newborns and in childhood and thus contributed to both analyses.

### DNAm and parent-reported sleep duration

**Fig. 1:**
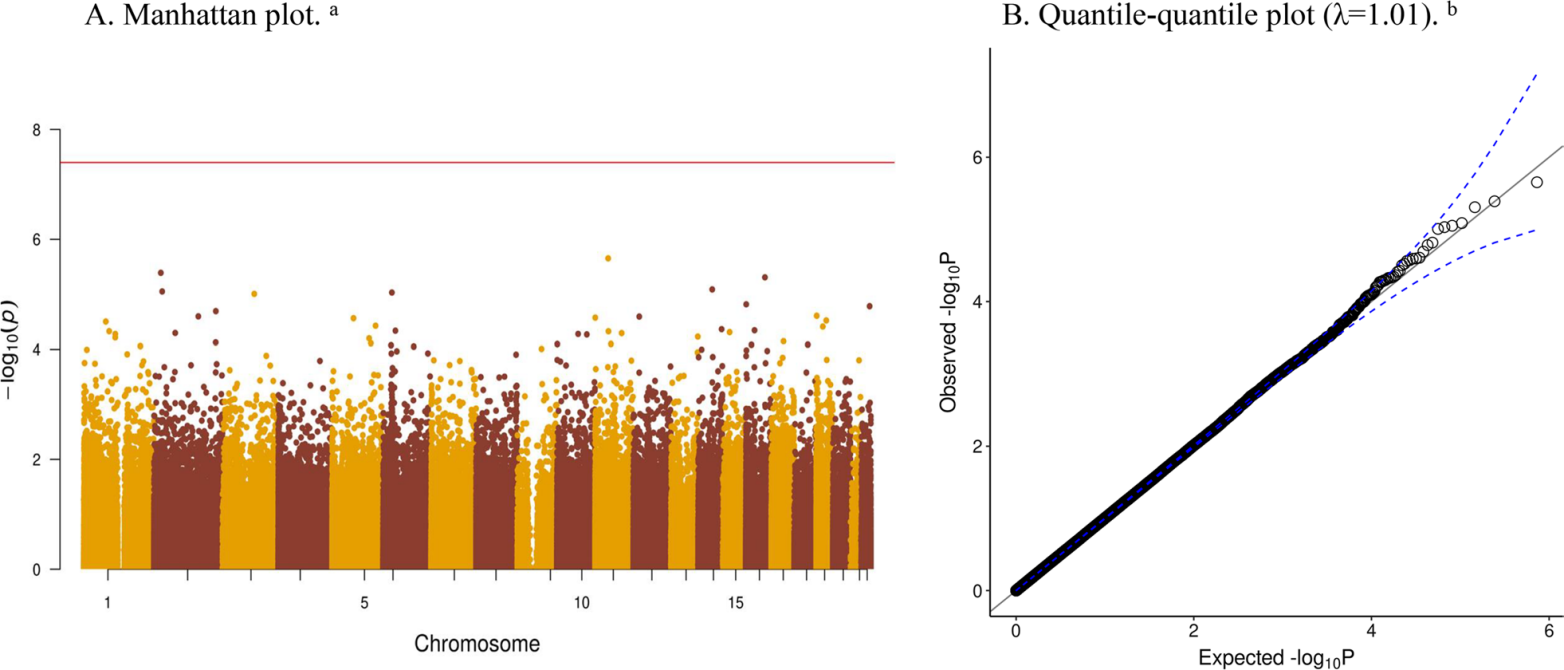
Newborn DNAm at birth and parent-reported child sleep duration among 3658 school-aged children. **A** Manhattan plot^a^. **B** Quantile–quantile plot (*λ* = 1.01)^b^. ^a^*Panel A* is a Manhattan plot: the x-axis shows the location of the CpG site in the genome, and the y-axis shows the − log_10_(p) of the observed meta-analytical association between DNAm at this CpG at birth and parent-rated sleep duration in childhood. The red line corresponds to the cut-off of statistical significance after multiple testing correction (4.0 × 10^–8^). ^b^*Panel B* is a quantile–quantile plot that shows the distribution of observed p values, compared to the distribution expected by chance

We did not observe associations between newborn DNAm and parent-reported child sleep duration in our primary meta-analysis among 3,658 children and a total of 364,672 CpGs, when correcting for multiple testing (cut-off p < 4.0 × 10^–8^) (Fig. 1A). We found no evidence of genomic inflation (λ = 1.01, quantile–quantile plot in Fig. 1B).

In sensitivity analyses, we repeated the primary meta-analysis among (1) European cohorts only, and (2) among cohorts with a mean age at sleep assessment ≥ 7 years (i.e. school-aged children), to harmonize the phenotype and reduce noise. We also repeated the analyses (3) with DNAm measured in *childhood*, rather than at birth, as our exposure variable, and reran the primary meta-analysis using (4) a sample-size-weighted, *p* value-based meta-analysis approach (that does not assume effect magnitude to be similar across cohorts) as an alternative to inverse variance-weighted fixed-effects meta-analysis. In line with the primary meta-analysis, none of these sensitivity analyses suggested any associations between DNAm and parent-reported sleep duration (Additional file 1: Table S3).

In Additional file 1: Table S3, we describe the subthreshold findings that came closest to statistical significance (i.e. the 25 CpG sites with a p value below the suggestive threshold of p < 5 × 10^–5^). For these subthreshold findings, we show the site-level results from the primary meta-analysis (of newborn DNAm and parent-reported sleep duration), and from analyses where we excluded non-European cohorts, excluded cohorts with child mean age < 7 years, used child (rather than newborn) DNAm as the exposure, or used actigraphy-estimated (rather than parent-reported) sleep duration as the outcome. Briefly, for only 5 out of 25 sites, all cohorts reported a consistent direction of effects in the primary meta-analysis: lower methylation of *cg14340131* [annotated to *HRAS*] and higher methylation of *cg01532396* [not annotated to any genes], *cg04384689* [not annotated to any genes], *cg10143030* [*WDR43;SNORD92*] and *cg17853707* [*ZNF91*]. One of these sites, *cg01532396* was the CpG that came closest to statistical significance in the meta-analysis (*p* = 2.22 × 10^–6^). We also investigated if any of these 25 subthreshold findings were located nearby (< 1Mbase) 172 single-nucleotide polymorphisms (SNP) that have previously been associated with sleep duration (https://sleep.hugeamp.org, accessed 2021/06/23). Only one CpG, *cg24769432*, was located within a 1Mbase region of a sleep-related SNP, *rs4538155* (*LINC01876* gene located in 2q24.1). Cohort-level results showed inconsistent directions of effect for associations between DNAm at this CpG and sleep duration. Further, none of the 25 subthreshold findings from the primary meta-analysis overlapped with any subthreshold findings (*p* < 5 × 10^–5^) from our other (secondary) meta-analyses of newborn DNAm and parent-reported sleep initiation or fragmentation problems.

### DNAm and parent-reported sleep initiation and fragmentation problems

We observed no associations between newborn DNAm and parent-reported child sleep initiation (Additional file 1: Figure S2) or fragmentation (Additional file 1: Figure S3) problems among 2,504 and 1,681 children, respectively. We repeated the analyses using DNAm measured at *childhood*, rather than at birth: again, no associations were observed (Additional file 1: Figure S2–S3).

### DNAm and actigraphy-estimated sleep outcomes

Newborn DNAm and actigraphy-estimated sleep data were available among 582 school-aged children from 3 cohorts. Additional file 1: Figure S4, S5 and S6 show meta-analytic associations between newborn DNAm and actigraphy-estimated sleep duration, sleep onset latency (time between lying down in bed and falling asleep) and wake-after-sleep-onset duration (time awake between falling asleep and final awakening), respectively.

We identified two statistically differentiated CpGs (*p* < 4.0 × 10^–8^) in analyses of newborn DNAm and child sleep, and none in the analyses of DNAm in childhood and child sleep. These two hits are described briefly below: for details, see Additional file 1: Table S4. First, lower cord blood DNAm at *cg24815001* was associated with longer *actigraphy-estimated sleep duration* among 577 children (*p* = 3.31 × 10^–8^). The cohort-level direction of effects was negative in all three cohorts with data, and the meta-analytic effect estimate corresponded to a 0.69-SD-unit reduction in sleep duration z-scores per 10% increase in methylation (i.e. effect estimate − 6.9 per change from completely non-methylated (0) to completely methylated (1), standard error 1.2, *I*^2^ = 0). In our study, *childhood* DNAm at *cg24815001* was not associated with actigraphy-based sleep duration (*p* = 0.84), nor was newborn cord blood DNAm at *cg24815001* associated with *parent-reported* sleep duration (*p* = 0.96). We are not aware of previous EWAS studies linking *cg24815001,* an open-sea CpG in chromosome 7 with child (or adult) phenotypes (http://www.ewascatalog.org/?cpg=cg24815001, accessed 2021/06/09), nor is it annotated to any genes [35]. At *cg24815001*, blood cell DNAm is only weakly correlated with DNAm in brain tissues (*r* < 0.06) (https://epigenetics.essex.ac.uk/bloodbrain/?probenameg=cg24815001, accessed 2021/06/09).

Second, lower cord blood DNAm at *cg02753354* was associated with longer actigraphy-estimated sleep onset latency among 580 children (*p* = 8.8 × 10^–9^). However, heterogeneity between our three cohorts was considerable (*I*^2^ = 74.1). The results were driven by one cohort (Generation R): negative effect estimates were observed in Generation R (beta = − 11.9, *p* = 3.37 × 10^–9^, *n* = 257) and PROGRESS (beta = − 4.7, *p* = 0.16, *n* = 242), while INMA reported a positive effect estimate (beta = 7.7, *p* = 0.37, *n* = 81). Further, *childhood* DNAm of *cg02753354* was not associated with sleep onset latency (*p* = 0.85, *n* = 712). Newborn DNAm of *cg02753354* was not associated with *parent-reported* sleep initiation problems (*p* = 0.77, *n* = 1,712). This CpG in chromosome 19 is annotated to the *ARHGAP45* gene, which has a circadian expression profile (http://circadb.hogeneschlab.org/human, last accessed 2021/06/22). *ARHGAP45* codes for a precursor of the histocompatibility antigen HA-1 and a GTPase activator for the Rho-type GTPases (https://www.genecards.org/cgi-bin/carddisp.pl?gene=ARHGAP45&keywords=hmha1, last accessed 2021/06/09) and has been linked to endothelial integrity and immune cell maturation [48, 49]. In a previous study investigating the associations between DNA co-methylation modules and sleep in one of the cohorts included in this meta-analysis, namely Generation R, authors reported that a module containing a CpG located at *ARHGAP27* was associated with actigraphy-assessed sleep duration [14]. Additionally, a genome-wide association study based on UK Biobank and 23andMe data indicated that variants in *ARHGAP27* were associated with self-reported sleep traits, including sleep duration [50]. In previous epigenetic studies, lower cord blood DNAm at *cg02753354* has been linked to higher birth weight [51] and gestational age at birth [52, 53], and a cross-sectional adult study suggested an association with ischaemic stroke [54]. However, correlations between *cg02753354* DNAm in blood vs another (perhaps more relevant) target tissue, the brain, are weak (*r* < 0.11) [42].

### Gene ontology (GO) enrichment

We performed gene ontology (GO) pathway analyses on genes annotated to CpGs with the lowest *p* values (*p* < 1.0 × 10^–4^) (see *Methods* for details). GO term enrichment analyses did not suggest any GO terms to be over- or underrepresented within the gene set of interest in the primary or secondary meta-analyses.

### Differentially methylated region (DMR) analyses

We used two alternative approaches (*DMRcate* and *ipDMR*) to identify DMRs in cord blood, with all six child sleep phenotypes. The results are shown in Additional file 1: Table S5. Neither approach identified any DMRs associated with parent-reported child sleep duration, initiation, or fragmentation.

*DMRcate* yielded 1 DMR including 6 CpGs (chromosome 11: start–end 2,292,890–2,293,048 [annotated to *ASCL2*]) associated with actigraphy-estimated sleep onset latency. On the other hand, *ipDMR* yielded 8 regions associated with actigraphy-estimated sleep phenotypes, 6 of which included at least two CpGs: sleep onset latency was associated with 3 DMRs (chr19:1,074,425–1,074,927 [*ARHGAP45*]; chr6:56,819,612–56,819,616 [*BEND6;DST*]; and chr17:46,669,566–46,669,645 [*LOC404266;HOXB5*]), and wake-after-sleep-onset duration with 3 DMRs (chr19:48,894,694–48,894,716 [*KDELR1*]; chr4:187,422,114–187,422,120 [not annotated to genes]; and chr6:33,048,254–33,048,287 [*HLA-DPB1*]). None of these DMRs identified using two alternative tools (*DMRcate, ipDMR*) showed any overlap.

## Discussion

This meta-analysis did not identify associations between DNAm at birth and parent-reported sleep duration among 3,658 children. Similarly, we found no associations between DNAm at birth and parent-reported sleep initiation or fragmentation problems.

Actigraphy can provide a more objective and potentially more sensitive measure of child sleep duration compared to parental reports [55, 56]. Actigraphy can also be used to measure sleep onset latency, an indicator of sleep initiation problems, and wake-after-sleep-onset duration, which can increase when the number of sleep fragments or the average time spent awake between fragments (or both) increases. However, collecting actigraphy data is often relatively cumbersome, compared to questionnaire-based data. The current meta-analysis brought together three cohorts that have both DNAm at birth and actigraphy-estimated child sleep data. Their results showed that lower DNAm at *cg24815001* and *cg02753354* at birth was associated with longer actigraphy-estimated sleep duration (*n* = 577) and longer actigraphy-estimated sleep onset latency (*n* = 580) in childhood, respectively. It is possible that actigraphy-estimated sleep assessments were more sensitive to picking up differences in child sleep that relate to DNAm than parental reports. However, due to the small number of participants and, in the case of *cg02753354*, heterogeneity between cohorts, we advise caution in accepting these findings as proof of underlying epigenetic signatures or mechanisms without further studies. Further, we identified some potentially differentially methylated regions in relation to actigraphy-estimated sleep onset latency and wake-after-sleep-onset duration, yet the small number of cohorts and the failure to replicate the findings using two alternative tools raise some doubt on whether these reflect meaningful differences in DNAm. Both individual CpG and region-level findings should be further studied in larger paediatric cohorts, should such large-scale data become available.

Our results do not provide consistent evidence that DNAm from blood at birth or in childhood would explain differences in child sleep, neither from parent-reported outcomes, nor actigraphy-based data. Alternative mechanisms that can affect inter-individual differences in child sleep characteristics include, for example, family bedtime practices, socio-economic disparities and genetic make-up [57]. While the results of this large meta-analysis may be less vulnerable to false positives than the limited number of prior smaller, single-sample studies, it is also possible that discrepancies between our and previous studies reflect true biological phenomena. Most importantly, our study focussed on prospective associations with child sleep, while previous studies have mostly studied DNAm and sleep among adults (e.g. [11–13]). In a cross-sectional design, DNAm could reflect a biomarker of poor sleep, rather than a causal factor underlying variation in sleep. Further, sleep could be differentially restricted by external factors during childhood compared to adulthood, which in turn could affect the extent of observable epigenetically driven variance. It would be interesting to see if large-scale epigenome-wide studies that span to adolescence and adulthood could establish differences that emerge at this later stage.

DNAm at nearby sites is correlated, and there is disagreement over the optimal method of genome-wide correction. In the current study, however, the findings of the primary meta-analysis would have remained the same (in this case, null) if we had ignored the additional tests introduced by the secondary meta-analyses completely and only applied the more lenient Safari cut-off (2.4 × 10^–7^), [33] or if we had used the Bonferroni cut-off that assumes total independence of exposures and outcomes (0.05/364,672/6 = 2.3 × 10^–8^).

The current study has several strengths. This meta-analysis of previously unpublished EWAS results from a total of 14 cohorts is the largest epigenetic study on child sleep to date. It is extensive in terms of both sample size and the scope of the epigenome-wide rather than candidate site approach. The harmonized, predefined analysis plan increases comparability across studies, while the collaboration between many independent studies improves reliability and generalizability of the results. The variety of sleep outcomes provide a window into several important aspects of child sleep, both objective and subjective. Finally, the inclusion of DNAm data at both birth and in childhood accounts for the partial instability of DNAm across childhood [32] and can elucidate temporal relations beyond the scope of cross-sectional designs.

Our study also has limitations. First, while this is the largest study so far, it is possible we are still underpowered to identify subtle associations between DNAm and sleep. Further, differences between cohorts, relating for example to age at assessment and cultural and genetic differences between populations, can obscure associations that are specific to certain subgroups, and differences in how sleep was measured (e.g. phrasing of questionnaires) could introduce noise. Second, we combined the 450 k and EPIC array data sets, which could also introduce some noise; however, correlations between these arrays are high for DNAm in blood [29]. Third, we used parent-reported sleep measures as primary outcomes because of comparability across studies, large sample size, and previously reported associations with genetic and early-life environmental factors. However, these measurements are prone to misclassification and may have contributed measurement error to our analyses [55]. We attempted to overcome this limitation by investigating actigraphy-estimated sleep measures as more objective measures, when available. Fourth, we measured DNAm in peripheral blood and not in the brain. Beyond a potential mechanism, blood DNAm could be interpreted as an early-life marker of causal genetic, biological or environmental influences on sleep, thus potentially lending new insights into factors shaping sleep outcomes, rather than biological mechanism. Future research, including advanced causal inference tools and molecular research, would be necessary to establish whether identified hits in blood DNAm are the causal mediator or a proxy of other causal drivers.

Even though we found no consistent evidence of differential methylation related to child sleep, we encourage future research into sleep phenotypes that were beyond the scope of the current study. For example, clinical studies could identify epigenetic patterns associated with clinically diagnosed sleep disorders, which population-based studies may not capture. Future studies should also consider longitudinal associations between DNAm and child sleep. Repeated methylation assessment with concurrent sleep assessments would give insight into trajectories of changes in DNA methylation over time and their long-term effects on sleep. Further, epigenetic mechanisms such as histone modifications or effects limited to specific target tissues (e.g. neuroendocrine tissues) could be of interest, if large-scale analyses of these mechanisms become possible in the future.

In conclusion, we found no consistent evidence of an association between cord blood or peripheral blood DNAm and sleep among children. Larger studies or studies that focus to overcome some of our limitations could reveal subtle associations or confirm associations limited to objectively measured sleep outcomes.

## Supplementary Information

**Additional file1: Table S1.** Characteristics of the participating cohorts in analyses of DNAm in childhood and child sleep outcomes.** Table S2.** Overlap between cord blood and peripheral blood in childhood DNA methylation analyses.** Table S3.** Site-specific results for the 25 CpGs that came closest to statistical significance (*p*<5.0×10-5) in the primary meta-analysis of DNAm at birth and parent-reported sleep duration in school age.** Table S4.** Secondary meta-analyses: significant associations between DNAm and child sleep (*p*<4.0×10-8).** Table S5.** Analyses of differentially methylated regions (DMRs) in cord blood at birth and child sleep.** Figure S1.** Correlations and independence of the six phenotypes of interest.** Figure S2.** DNAm and parent-reported child sleep initiation problems among school-aged children: Manhattan and quantile–quantile plots.** Figure S3.** DNAm and parent-reported child sleep fragmentation problems among school-aged children: Manhattan and quantile–quantile plots.** Figure S4.** DNAm and actigraphy-estimated child sleep duration among school-aged children: Manhattan and quantile–quantile plots.** Figure S5.** DNAm and actigraphy-estimated child sleep onset latency among school-aged children: Manhattan and quantile–quantile plots.** Figure S6.** DNAm and actigraphy-estimated child wake-after-sleep-onset duration among school-aged children: Manhattan and quantile–quantile plots.

**Additional file2.** Methods

## Data Availability

Site-level meta-analytical results are available at the EWAS Catalog *[all site-level results from the primary and secondary meta-analyses will be publicly uploaded through EWAS Catalog, *http://www.ewascatalog.org/*, and link will be added upon acceptance or according to journal embargo policy].* For access to cohort-level data, requests can be sent directly to individual studies.
