# Supplementary material for: Longitudinal associations of DNA methylation and sleep in children: a meta-analysis
Source: Clin Epigenetics. 2022 Jul 5;14:83. doi: 10.1186/s13148-022-01298-4 (PMC9258202; doi:10.1186/s13148-022-01298-4)
Supplement: Supplementary file 1 — Additional file1: Table S1. Characteristics of the participating cohorts in analyses of DNAm in childhood and child sleep outcomes. Table S2. Overlap between cord blood and peripheral blood in childhood DNA methylation analyses. Table S3. Site-specific results for the 25 CpGs that came closest to statistical significance (p<5.0×10^-5^) in the primary meta-analysis of DNAm at birth and parent-reported sleep duration in school age. Table S4. Secondary meta-analyses: significant associations between DNAm and child sleep (p<4.0×10^-8^). Table S5. Analyses of differentially methylated regions (DMRs) in cord blood at birth and child sleep. Figure S1. Correlations and independence of the six phenotypes of interest. Figure S2. DNAm and parent-reported child sleep initiation problems among school-aged children: Manhattan and quantile–quantile plots. Figure S3. DNAm and parent-reported child sleep fragmentation problems among school-aged children: Manhattan and quantile–quantile plots. Figure S4. DNAm and actigraphy-estimated child sleep duration among school-aged children: Manhattan and quantile–quantile plots. Figure S5. DNAm and actigraphy-estimated child sleep onset latency among school-aged children: Manhattan and quantile–quantile plots. Figure S6. DNAm and actigraphy-estimated child wake-after-sleep-onset duration among school-aged children: Manhattan and quantile–quantile plots. [file 13148_2022_1298_MOESM1_ESM.pdf]

Longitudinal associations of DNA methylation and sleep in children: A meta-analysis

Supplementary Tables and Figures

Contents:

Table S1. Characteristics of the participating cohorts in analyses of DNAm *in childhood* and child sleep outcomes
Table S2. Overlap between cord blood and peripheral blood in childhood DNA methylation analyses
Table S3. Site-specific results for the 25 CpGs that came closest to statistical significance ($p<5.0\times10^{-5}$) in the primary meta-analysis of DNAm at birth and parent-reported sleep duration in school age
Table S4. Secondary meta-analyses: significant associations between DNAm and child sleep ($p<4.0\times10^{-8}$)
Table S5. Analyses of Differentially Methylated Regions (DMRs) in cord blood at birth and child sleep.
Figure S1. Correlations and independence of the six phenotypes of interest.
Figure S2. DNAm and parent-reported child sleep initiation problems among school-aged children: Manhattan and quantile-quantile plots.
Figure S3. DNAm and parent-reported child sleep fragmentation problems among school-aged children: Manhattan and quantile-quantile plots.
Figure S4. DNAm and actigraphy-estimated child sleep duration among school-aged children: Manhattan and quantile-quantile plots.
Figure S5. DNAm and actigraphy-estimated child sleep-onset-latency among school-aged children: Manhattan and quantile-quantile plots.
Figure S6. DNAm and actigraphy-estimated child wake-after-sleep-onset duration among school-aged children: Manhattan and quantile-quantile plots.

Table S1. Characteristics of the participating cohorts in analyses of DNAm in childhood and child sleep outcomes

	**ALSPAC**	**CHOP**	**EDEN**	**Generation R**	**GLAKU**	**HELIX**	**INMA**	**Viva**
Country	UK	Europe [a]	France [b]	Netherlands	Finland	Europe [b]	Spain [b]	USA
Children with DNAm in childhood and sleep data, n	917	308	123	405	201	932	50	438
DNAm array type for child blood	450K	450K	450K	450K	EPIC	450K	450K	450K
Child age at venepuncture (years), mean (SD)	7.4 (0.1)	11.1 (0.1)	5.7 (0.1)	9.8 (0.3)	12.4 (0.5)	7.7 (1.7)	4.4 (0.1)	7.8 (0.8)
Parent-reported child sleep								
Number of children with data, n (%)	813 [c]	0	123	155 [d]	201	932	0	438
Child age at assessment, years, mean (SD)	11.7 (0.1)	*n/a*	5.7 (0.1)	9.7 (0.3)	12.4 (0.5)	7.7 (1.7)	*n/a*	7.8 (0.8)
Duration, hours per day, mean (SD)	9.9 (0.6)	*n/a*	*n/a*	9.5 (0.7)	9.2 (0.8)	10.2 (0.7)	*n/a*	9.7 (0.9)
Initiation difficulties, yes, n (%)	457 (52.1)	*n/a*	46 (37.4)	83 (18.8)	30 (14.9)	*n/a*	*n/a*	*n/a*
Sleep fragmentation, yes, n (%)	91 (10.6)	*n/a*	43 (35.0)	*n/a*	50 (24.9)	*n/a*	*n/a*	*n/a*
Actigraphy-based child sleep								
Number of children with data, n (%)	0	308	0	158	201	0	50	0
Child age at assessment, years, mean (SD)	*n/a*	11.1 (0.1)	*n/a*	11.7 (0.1)	12.4 (0.5)	*n/a*	11.1 (0.5)	*n/a*
Duration (total sleep time), hours, mean (SD)	*n/a*	8.9 (0.7)	*n/a*	7.6 (0.7)	9.0 (0.5)	*n/a*	7.2 (0.9)	*n/a*
Sleep onset latency, minutes, mean (SD)	*n/a*	10.7 (6.8)	*n/a*	62.4 (40.8)	20.8 (14.2)	*n/a*	6.8 (16.8)	*n/a*
Wake after sleep onset duration, minutes, mean (SD)	*n/a*	98.3 (41.2)	*n/a*	90.3 (28.9)	30.1 (7.1)	*n/a*	40.5 (23.1)	*n/a*
Maternal characteristics								
Education, low, n (%) [e]	442 (49.1)	51 (16.6)	29 (23.6)	36 (8.1)	35 (17.4)	169 (18.1)	12 (24.0)	11 (2.5)
Age, years, mean (SD)	30.1 (4.4)	32.3 (4.4)	30.3 (5.0)	32.2 (4.0)	30.4 (4.4)	30.3 (5.0)	30.8 (4.4)	32.0 (5.7)
Smoking during pregnancy, n (%)								
No smoking during pregnancy	782 (87.2)	248 (80.5)	94 (76.4)	319 (77.6)	182 (90.5)	713 (76.5)	40 (80.0)	387 (88.3)
Smoked during pregnancy	115 (12.8)	60 (19.5)	29 (23.6)	92 (22.4)	19 (9.5)	219 (23.5)	10 (20.0)	51 (11.6)
Quit in early pregnancy	27 (3.0)	13 (4.2)	9 (7.3)	44 (10.7)	*n/a*	*n/a*	5 (10.0)	*n/a*
Continued smoking	88 (9.8)	47 (15.3)	20 (16.3)	48 (11.7)	*n/a*	*n/a*	5 (10.0)	*n/a*
Child characteristics								
Sex, female, n (%)	465 (50.7)	167 (54.2)	52 (42.3)	219 (49.5)	108 (53.7)	422 (45.3)	22 (44.0)	214 (48.9)
Gestational age, weeks, mean (SD)	39.6 (1.5)	n/a	39.4 (1.5)	40.1 (1.6)	40.0 (1.3)	39.5 (1.6)	39.6 (1.5)	39.5 (1.6)

Abbreviations: 450K: Illumina Infinium® HumanMethylation450 BeadChip; DNAm: deoxyribonucleic acid methylation; EPIC: Illumina Infinium® HumanMethylationEPIC BeadChip; ISCED: International Standard Classification of Education, kg: kilogram; n: number of participants; n/a: not applicable due to lack of available data; SD: standard deviation; UK: United Kingdom; USA: United States of America

[a] CHOP and HELIX are international multi-center cohorts. CHOP includes participants from Germany, Belgium, Italy, Poland, and Spain.

[b] HELIX includes participants from Spain, France, United Kingdom, Lithuania, and Greece. EDEN and INMA are also part of HELIX, as explained in more detail in the Supplementary Methods. HELIX had data available on parent-reported sleep duration and DNAm in childhood, and ran individual-participant pooled EWAS on these data. For actigraphy-based sleep characteristics, data were only available in one HELIX cohort (INMA) who thus ran these analyses within INMA only. For sleep initiation and sleep fragmentation, data were only available in one HELIX cohort (EDEN) who thus ran these analyses within EDEN only. Due to the small number of participants with parent-reported sleep initiation and fragmentation problems among the INMA participants with childhood DNAm, these aspects of child sleep could not be analyses in relation to childhood DNAm in INMA.

[c] In ALSPAC, sleep outcomes were collected at different follow-ups, and sample size and age varied per outcome: of those with DNAm data in childhood, 813 children had data on parent-reported sleep duration at the mean age of 11.7 years (SD=0.1), while 853 and 832 children had data on parent-reported sleep initiation and fragmentation problems at the age of 9.6 years (SD=0.1), respectively.

[d] In Generation R, sleep outcomes were collected at different follow-ups, sample size and age varied per outcome: of those with DNAm data in childhood, 155 children had data on parent-reported sleep duration at the mean age of 11.7 years (SD=0.2), while 405 children had data on parent-reported sleep initiation problems at the mean age of 9.7 years (SD=0.3).

[e] Rates of low education are not directly comparable, as educational systems differed between countries and cohorts used different definitions of low education, as explained in more detail in Supplementary Methods.

Table S2. Overlap between cord blood and peripheral blood in childhood DNA methylation analyses

	ALSPAC	CHOP	EDEN	Generation R	GLAKU	Healthy Start	HELIX	INMA	LINA	MoBa-1	MoBa-2	PREDO	PROGRESS	Viva
Country	UK	Europe [a]	France [b]	Netherlands	Finland	USA	Europe [b]	Spain [b]	Germany	Norway	Norway	Finland	Mexico	USA
Children with DNAm in childhood and sleep data, n	917	308	123	405	201	0	932	50	0	0	0	0	0	438
Children with DNAm at birth and sleep data, n	855	0	122	597	0	283	0	260	204	711	415	247	244	410

Abbreviations: n: number of participants; UK: United Kingdom; USA: United States of America

[a] CHOP and HELIX are international multi-center cohorts. CHOP includes participants from Germany, Belgium, Italy, Poland, and Spain.

[b] HELIX includes participants from Spain, France, United Kingdom, Lithuania, and Greece. EDEN and INMA are also part of HELIX, as explained in more detail in the Supplementary Methods. HELIX had data available on parent-reported sleep duration and DNAm in childhood, and ran individual-participant pooled EWAS on these data. For actigraphy-based sleep characteristics, data were only available in one HELIX cohort (INMA) who thus ran these analyses within INMA only. For sleep initiation and sleep fragmentation, data were only available in one HELIX cohort (EDEN) who thus ran these analyses within EDEN only. Due to the small number of participants with parent-reported sleep initiation and fragmentation problems among the INMA participants with childhood DNAm, these aspects of child sleep could not be analyses in relation to childhood DNAm in INMA.

Table S3. Site-specific results for the 25 CpGs that came closest to statistical significance (p<5E-05) in the primary meta-analysis of DNAm at birth and parent-reported sleep duration in school age

**Description of the CpG**							**DNAm at birth and parent-reported sleep duration in school age: *Primary meta-analysis results***							
					**Relation to CpG island**					**Heterogeneity**		**Sample size**		
**CpG**	**Chr**	**Position**	***Annotated gene***	***Located near sleep-related SNP***	***Relation***	***Related island***	**Beta**	**SE**	**P-value**	***I^2***	***P-value***	***Cohorts***	***Children***	**Effect direction per cohort** [e]
**cg01532396**	11	46265134	*n/a*	*no*	Island	chr11:46264973-46265335	12.1	2.6	2.22E-06	8	0.37	10	3640	++++++++++
**cg27418895**	2	24272716	*FKBP1B*	*no*	Island	chr2:24272582-24273313	6.0	1.3	4.08E-06	0	0.83	10	3645	-+++++++++
**cg10368536**	16	67518179	*AGRP*	*no*	S shelf	chr16:67514398-67515345	-3.9	0.8	4.93E-06	55	0.02	10	3641	-+-------+
**cg26142058**	14	70193981	*n/a*	*no*	S shore	chr14:70193467-70193955	7.4	1.7	8.18E-06	25	0.22	10	3631	++++-++++-
**cg10143030**	2	29135532	*WDR43;SNORD92*	*no*	Open sea	*n/a*	5.7	1.3	8.90E-06	0	0.45	10	3626	++++++++++
**cg03625897**	6	31587864	*BAT2*	*no*	Island	chr6:31587779-31589024	-14.4	3.2	9.31E-06	2	0.42	9	3377	-------+?+
**cg00173906**	3	113000000	*n/a*	*no*	Open sea	*n/a*	-3.2	0.7	9.85E-06	0	0.78	10	3622	---+-----+
**cg27616039**	16	1207075	*CACNA1H*	*no*	Island	chr16:1207042-1208032	-9.0	2.1	1.53E-05	0	0.46	10	3595	------+---
**cg25060632**	22	46683277	*TTC38*	*no*	N shelf	chr22:46685379-46685796	11.9	2.8	1.65E-05	0	0.91	10	3608	+-++++++++
**cg07028390**	2	219000000	*RQCD1;USP37*	*no*	Island	chr2:219432767-219434061	7.9	1.9	2.03E-05	0	0.85	10	3644	+-++-++++-
**cg25246158**	19	1940184	*CSNK1G2*	*no*	N shore	chr19:1940303-1941940	3.9	0.9	2.47E-05	43	0.07	10	3611	+++++-+-+-
**cg24769432**	2	157000000	*GPD2*	*yes: rs4538155* [j]	Open sea	*n/a*	-2.3	0.5	2.53E-05	51	0.03	10	3622	-+------++
**cg03833948**	12	21678044	*C12orf39*	*no*	N shelf	chr12:21680408-21680982	-1.6	0.4	2.55E-05	0	0.75	10	3623	--+-------
**cg14340131**	11	533457	*HRAS*	*no*	N shore	chr11:534691-537718	-2.6	0.6	2.67E-05	0	0.98	10	3636	----------
**cg22581187**	5	75966681	*IQGAP2*	*no*	Open sea	*n/a*	1.4	0.3	2.73E-05	0	0.97	10	3656	+-++++++++
**cg27017562**	19	35782899	*MAG*	*no*	N shelf	chr19:35786551-35786962	-2.8	0.7	3.00E-05	33	0.15	10	3651	-+--++-+--
**cg03000585**	1	78355339	*NEXN*	*no*	S shore	chr1:78354381-78354673	-1.5	0.4	3.14E-05	0	0.54	10	3655	-----+----
**cg20776454**	5	154000000	*CNOT8*	*no*	Island	chr5:154237008-154238362	12.8	3.1	3.74E-05	44	0.06	10	3641	+++++++-++
**cg17853707**	19	23578260	*ZNF91*	*no*	Open sea	*n/a*	6.9	1.7	3.87E-05	0	0.93	10	3651	++++++++++
**cg13148496**	14	103000000	*CDC42BPB*	*no*	Island	chr14:103430534-103430787	-7.5	1.8	4.32E-05	46	0.05	10	3629	-+-++-----
**cg00966098**	16	30596551	*ZNF785*	*no*	Island	chr16:30596487-30596900	6.3	1.5	4.52E-05	0	0.77	10	3645	++++-++---
**cg04384689**	6	44039689	*n/a*	*no*	N shore	chr6:44041014-44041468	5.6	1.4	4.61E-05	0	0.76	10	3637	++++++++++
**cg09270879**	1	90405538	*n/a*	*no*	Open sea	*n/a*	-6.1	1.5	4.70E-05	45	0.06	10	3632	-----+---+
**cg12565914**	11	47270322	*ACP2;NR1H3*	*no*	Island	chr11:47270234-47270633	-6.8	1.7	4.71E-05	0	0.67	10	3628	-------++-
**cg27649764**	15	44719030	*CTDSPL2*	*no*	N shore	chr15:44719186-44720138	-11.6	2.9	4.89E-05	0	0.52	10	3631	-------+--

(continued on next page)

(continued from previous page)

	**DNAm at birth and parent-reported sleep duration in school age:** *Sensitivity meta-analysis among European cohorts* [a]							**DNAm at birth and parent-reported sleep duration among 7-12-year olds:** *Sensitivity meta-analysis among cohorts with mean age ≥7 years* [b]						
					**Sample size**							**Sample size**		
**CpG**	**Beta**	**SE**	**P-value**	I^2	*Cohorts*	*Children*	**Effect direction per cohort** [f]	**Beta**	**SE**	**P-value**	I^2	*Cohorts*	*Children*	**Effect direction per cohort** [g]
**cg01532396**	10.2	2.7	2.05E-04	0	8	2948	++++++++	12.0	3.1	1.03E-04	0	7	2988	+++++++
**cg27418895**	5.2	1.6	1.66E-03	0	8	2952	-+++++++	6.0	1.6	1.85E-04	0	7	2993	-++++++
**cg10368536**	-3.9	0.9	6.89E-06	55	8	2952	-+------	-4.0	0.9	7.98E-06	60	7	2989	------+
**cg26142058**	5.7	2.2	9.48E-03	19	8	2942	+++-++++	4.0	2.4	9.93E-02	12	7	2979	++-+++-
**cg10143030**	5.6	1.3	1.82E-05	18	8	2938	++++++++	6.2	1.4	1.25E-05	0	7	2974	+++++++
**cg03625897**	-13.7	3.6	1.29E-04	0	7	2684	------+?	-12.4	3.7	7.99E-04	13	7	2974	-----++
**cg00173906**	-3.4	0.7	2.98E-06	0	8	2934	--------	-3.4	0.8	1.16E-05	0	7	2971	------+
**cg27616039**	-8.7	2.1	4.75E-05	15	8	2904	-----+--	-8.5	2.2	1.41E-04	13	7	2945	----+--
**cg25060632**	12.4	2.9	1.99E-05	0	8	2920	+-++++++	12.9	3.0	1.64E-05	0	7	2960	+++++++
**cg07028390**	8.3	2.3	2.77E-04	0	8	2953	+-+-++++	7.8	2.6	3.00E-03	0	7	2992	++-+++-
**cg25246158**	4.5	1.0	4.69E-06	36	8	2923	++++-+-+	3.9	1.0	1.38E-04	61	7	2960	+++-+--
**cg24769432**	-2.5	0.6	8.98E-06	14	8	2935	-+-----+	-2.4	0.6	2.25E-05	50	7	2973	------+
**cg03833948**	-1.5	0.4	1.28E-04	0	8	2930	--+-----	-1.5	0.4	7.66E-05	0	7	2972	-+-----
**cg14340131**	-2.5	0.6	6.60E-05	0	8	2945	--------	-2.5	0.7	2.12E-04	0	7	2984	-------
**cg22581187**	1.4	0.4	3.93E-04	0	8	2963	+-++++++	1.4	0.4	5.36E-05	0	7	3004	+++++++
**cg27017562**	-2.8	0.7	1.68E-04	47	8	2963	-+-++-+-	-2.6	0.7	3.19E-04	51	7	3003	--++-+-
**cg03000585**	-1.5	0.4	9.85E-05	12	8	2963	----+---	-1.6	0.4	3.53E-05	14	7	3003	---+---
**cg20776454**	13.4	3.5	1.23E-04	49	8	2948	++++++-+	10.8	3.6	3.06E-03	20	7	2989	+++++-+
**cg17853707**	6.2	2.7	2.09E-02	0	8	2959	++++++++	6.2	2.8	2.53E-02	0	7	2999	+++++++
**cg13148496**	-7.4	1.9	6.75E-05	55	8	2936	-+-+----	-7.8	1.9	4.85E-05	42	7	2977	--+----
**cg00966098**	6.4	1.8	4.97E-04	0	8	2953	+++-++--	6.9	1.9	2.36E-04	0	7	3000	++-++--
**cg04384689**	5.6	1.4	8.45E-05	0	8	2947	++++++++	5.7	1.5	1.88E-04	0	7	2985	+++++++
**cg09270879**	-5.2	1.6	1.29E-03	44	8	2946	----+---	-5.0	1.7	4.02E-03	3	7	2982	---+--+
**cg12565914**	-6.6	2.0	7.73E-04	0	8	2940	------++	-7.4	1.9	8.68E-05	0	7	2977	-----+-
**cg27649764**	-11.0	3.1	3.70E-04	12	8	2940	------+-	-11.4	3.5	1.33E-03	14	7	2979	-----+-

(continued on next page)

(continued from previous page)

	**DNAm in school age and parent-reported sleep duration in school age:** *Sensitivity meta-analysis using DNAm measured in childhood* [c]							**DNAm at birth and actigraphy-estimated sleep duration in school age:** *Secondary meta-analysis using actigraphy-based sleep data* [d]						
					**Sample size**							**Sample size**		
**CpG**	**Beta**	**SE**	**P-value**	I^2	*Cohorts*	*Children*	**Effect direction per cohort** [h]	**Beta**	**SE**	**P-value**	I^2	*Cohorts*	*Children*	**Effect direction per cohort** [i]
**cg01532396**	-7.0	4.4	1.13E-01	32	4	2324	--?--	-10.6	5.3	4.33E-02	0	2	338	--?
**cg27418895**	-0.3	2.5	8.99E-01	0	4	2335	-+?-+	5.4	4.3	2.08E-01	0	2	338	+-?
**cg10368536**	1.1	1.0	2.52E-01	0	5	2532	+-+++	1.8	3.0	5.56E-01	55	3	582	++-
**cg26142058**	2.5	3.9	5.16E-01	0	5	2535	+-+-+	9.0	4.5	4.74E-02	0	3	580	+++
**cg10143030**	0.8	2.1	6.99E-01	43	5	2526	--+++	-1.6	3.2	6.04E-01	0	3	579	-++
**cg03625897**	5.6	4.4	2.02E-01	0	5	2532	-+-++	2.6	5.7	6.49E-01	0	3	572	++-
**cg00173906**	1.5	1.1	1.65E-01	25	5	2530	++++-	-0.8	2.1	6.98E-01	0	3	578	-++
**cg27616039**	-0.3	3.1	9.18E-01	0	5	2517	--+-+	-6.1	4.7	2.01E-01	0	3	578	-+-
**cg25060632**	3.8	4.6	4.03E-01	59	4	2331	+-?+-	-7.8	8.0	3.27E-01	27	2	337	--?
**cg07028390**	-2.1	4.6	6.59E-01	17	5	2535	+-+--	-9.2	4.8	5.42E-02	0	3	582	-++
**cg25246158**	-0.6	1.3	6.64E-01	43	5	2528	+--++	0.7	2.1	7.50E-01	0	3	575	+-+
**cg24769432**	-0.5	0.8	5.58E-01	0	5	2528	---+-	2.3	1.8	1.91E-01	0	3	569	+++
**cg03833948**	-0.4	0.4	3.12E-01	0	5	2525	-++++	-2.3	2.0	2.61E-01	0	3	581	-+-
**cg14340131**	0.5	0.7	4.35E-01	0	5	2533	+-+-+	2.8	2.3	2.23E-01	71	3	582	+-+
**cg22581187**	-0.4	0.4	3.70E-01	0	5	2539	--+--	-0.6	1.2	6.36E-01	30	3	582	-+-
**cg27017562**	-0.5	0.8	4.86E-01	0	5	2535	--+--	2.5	2.7	3.58E-01	0	3	581	+-+
**cg03000585**	-0.6	0.5	2.38E-01	31	5	2537	---+-	1.2	1.1	2.52E-01	0	3	582	+-+
**cg20776454**	1.8	4.5	6.89E-01	0	5	2537	-++++	9.5	6.1	1.19E-01	53	3	575	++-
**cg17853707**	-6.0	5.1	2.39E-01	0	5	2539	--+--	8.0	5.0	1.10E-01	0	3	581	+++
**cg13148496**	2.3	2.3	3.13E-01	0	5	2522	+++-+	-6.0	7.1	3.97E-01	0	3	581	---
**cg00966098**	-0.1	1.1	9.47E-01	0	4	2337	--?-+	-5.4	4.6	2.38E-01	4	2	338	--?
**cg04384689**	3.9	2.0	5.92E-02	0	5	2535	+++++	-0.8	3.4	8.16E-01	0	3	582	--+
**cg09270879**	-0.1	2.4	9.61E-01	0	5	2530	+-+-+	2.1	3.8	5.76E-01	69	3	579	--+
**cg12565914**	3.7	2.6	1.63E-01	23	5	2526	++-+-	0.5	4.2	9.08E-01	0	3	579	-++
**cg27649764**	0.7	4.8	8.80E-01	0	5	2528	++---	-11.4	5.8	4.86E-02	70	3	582	-+-

Table S4. Secondary meta-analyses: significant associations between DNAm and child sleep ($p<4.0\times10^{-8}$)

A. Description of the CpGs.

CpG	Description of the CpG: Chr	Position	Annotated gene	Relation to CpG island: *Relation*	*Related island*
**cg24815001**	7	33817425	*n/a*	Open sea	*n/a*
**cg02753354**	19	1074727	*HMHA1*	Island	chr19:1074726-1075071

B. Associations with sleep phenotypes.

CpG	DNAm at birth and actigraphy-estimated sleep duration in school age [a]							DNAm at birth and actigraphy-estimated sleep-onset-latency in school age [b]							DNAm in school age and actigraphy-estimated wake-after-sleep-onset time in school age [c]						
	Beta	SE	P-value	I^2	Sample size: *Cohorts*	Sample size: *Children*	Effect direction per cohort [d]	Beta	SE	P-value	I^2	Sample size: *Cohorts*	Sample size: *Children*	Effect direction per cohort [d]	Beta	SE	P-value	I^2	Sample size: *Cohorts*	Sample size: *Children*	Effect direction per cohort [d]
**cg24815001**	-6.9	1.2	3.31E-08	0	3	577	---	3.7	1.5	1.61E-02	0	3	577	+++	2.5	1.3	6.27E-02	61	3	569	+-+
**cg02753354**	0.8	1.5	5.74E-01	0	3	580	+++	-9.4	1.6	8.78E-09	74.1	3	580	-+-	-2.6	1.5	8.15E-02	0	3	580	-+-

Abbreviations: Beta: effect estimate from meta-analysis of linear regression model results, Chr: chromosome; DNAm: deoxyribonucleic acid methylation; I2: I squared, in which higher values reflect higher heterogeneity across studies; SE: standard error

[a] *cg24815001* was one the only CpG whose methylation was associated with actigraphy-based sleep duration in this meta-analysis ($p<4.0x10^{-8}$). The cohort-level effect estimate for this association was -6.6 in Generation R ($p=3.08x10^{-6}$, n=254), -1.2 in INMA (p=0.89, n=80), and 0.93 in PROGRESS (p=0.76, n=243).

[b] *cg02753354* was one the only CpG whose methylation was associated with actigraphy-based sleep-onset-latency in this meta-analysis ($p<4.0x10^{-8}$). The cohort-level effect estimate for this association was -11.9 in Generation R ($p=3.37x10^{-9}$, n=257), +7.7 in INMA (p=0.37, n=81), and -4.7 in PROGRESS (p=0.16, n=242).

[c] There were no associations between DNAm and actigraphy-based wake-after-sleep-onset duration in this meta-analysis ($p<4.0x10^{-8}$).

[d] Each sign denotes the direction of effects (+ positive, - negative, ? not available) per cohort, listed in the following (alphabetical order): Generation R, INMA, PROGRESS.

Table S5. Analyses of Differentially Methylated Regions (DMRs) in cord blood at birth and child sleep. [a]

A. DMRs identified using DMRcate. [b]

Phenotype	Chr	Start	End	CpGs	p	Annotated gene
Actigraphy-based sleep-onset-latency	11	2292890	2293048	6	1.52E-10	*ASCL2*

B. DMRs identified using ipDMR. [c]

Phenotype	Chr	Start	End	CpGs	p	Annotated gene
Actigraphy-based sleep duration	7	33817425	33817426	1	3.31E-08	*n/a*
Actigraphy-based sleep-onset-latency	19	1074425	1074927	3	7.41E-10	*HMHA1*
	6	56819612	56819616	2	1.92E-08	*BEND6;DST*
	6	160211790	160211791	1	5.71E-08	*TCP1;MRPL18*
	17	46669566	46669645	2	7.13E-07	*LOC404266; HOXB5*
Actigraphy-based wake-after-sleep-onset time	19	48894694	48894716	2	6.69E-09	*KDELR1*
	4	187422114	187422120	2	3.33E-07	*n/a*
	6	33048254	33048287	2	4.57E-07	*HLA-DPB1*

Abbreviations: Chr: chromosome; CpGs: number of CpGs within the region; DMR: Differentially Methylated Region; p: p-value after correction for multiple testing, i.e. minimum false-discovery-rate of the smoothed estimate from DMRcate or false-discovery-rate adjusted p-value from ipDMR

[a] Neither of the two methods (DMRcate, ipDMR) identified any DMRs associated with parent-reported child sleep duration (primary meta-analysis) or parent-reported child sleep initiation or fragmentation problems; all DMRs that were statistically significantly associated (after correction for multiple testing using false-discovery-rates) with any of the child phenotypes included in this study are presented in this table.

[b] DMRcate parameters were: lambda=500, C=5, min.cpgs=2, pcutoff="fdr"

[c] ipDMR parameters were: seed=0.05, dist.cutoff=1000, bin.size=50, include.all.sig.sites=TRUE

Figure S1. Correlations and independence of the six phenotypes of interest.

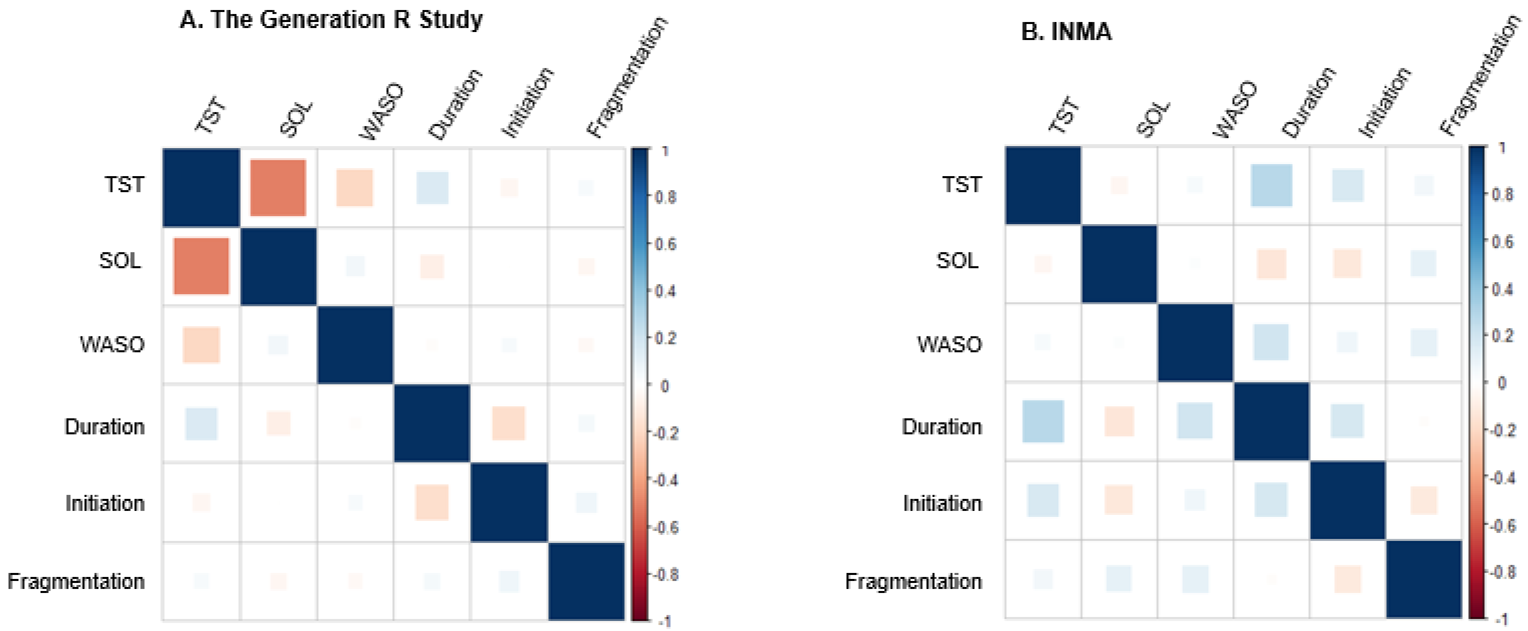

This figure depicts Spearman correlations between the six phenotypes in the two cohorts (Generation R, panel A; and INMA, panel B) that had data on all six phenotypes: overall, the correlations are low, supporting independence of the outcomes. We used the *meff* function from the *poolr* package in R to extracts eigenvalues from the individual-level matrix of phenotypic data to confirm the effective number of tests, which was estimated at 5-6 in both cohorts, depending on whether the approach recommended by Gao et al. (2008) (https://doi.org/10.1002/gepi.20310) or Galwey (2009) (https://doi.org/10.1002/gepi.20408) was taken.

Abbreviations: Duration: parent-reported sleep duration; Fragmentation: parent-reported sleep fragmentation problems; Initiation: parent-reported sleep initiation problems; SOL: actigraphy-based sleep-onset-latency; TST: actigraphy-based total sleep time (i.e. sleep duration); WASO: actigraphy-based wake-ofter-sleep-onset

Figure S2. DNAm and parent-reported child sleep initiation problems among school-aged children: Manhattan and quantile-quantile plots. [a]

A. Manhattan plot.

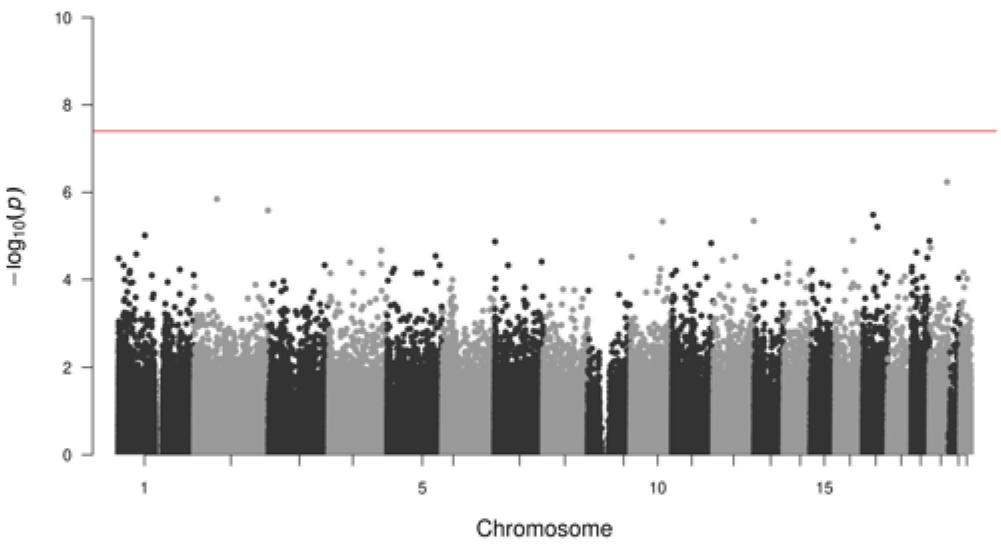

B. Quantile-quantile plot (lambda = 1.08).

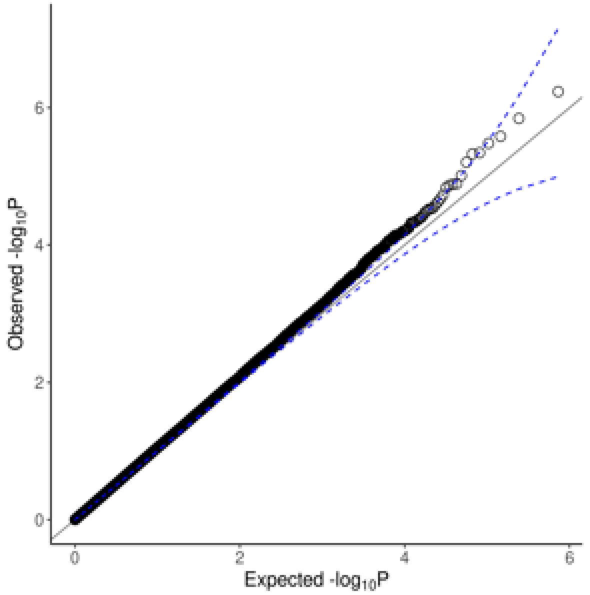

In panel A, the x axis shows the location of the CpG and the y axis shows the -log10(p) of the observed meta-analytical association between DNAm at this CpG at birth and parent-rated sleep initiation problems in childhood. The red line corresponds to the cut-off of statistical significance after multiple-testing correction ($p<4.0\times10^{-8}$). Panel B is a quantile-quantile plot that shows the distribution of observed p-values, compared to the distribution expected by chance.

[a] These plots depict associations between DNAm at *birth* and sleep initiation problems in childhood (n=2,504). In a sensitivity analysis, we re-ran the meta-analysis using results where DNAm in *childhood* was used to predict sleep initiation problems in childhood (n=1,582). The results remained unchanged: we observed no associations between DNAm and sleep initiation problems ($p<4.0\times10^{-8}$) (lambda=1.02).

Figure S3. DNAm and parent-reported child sleep fragmentation problems among school-aged children: Manhattan and quantile-quantile plots. [a]

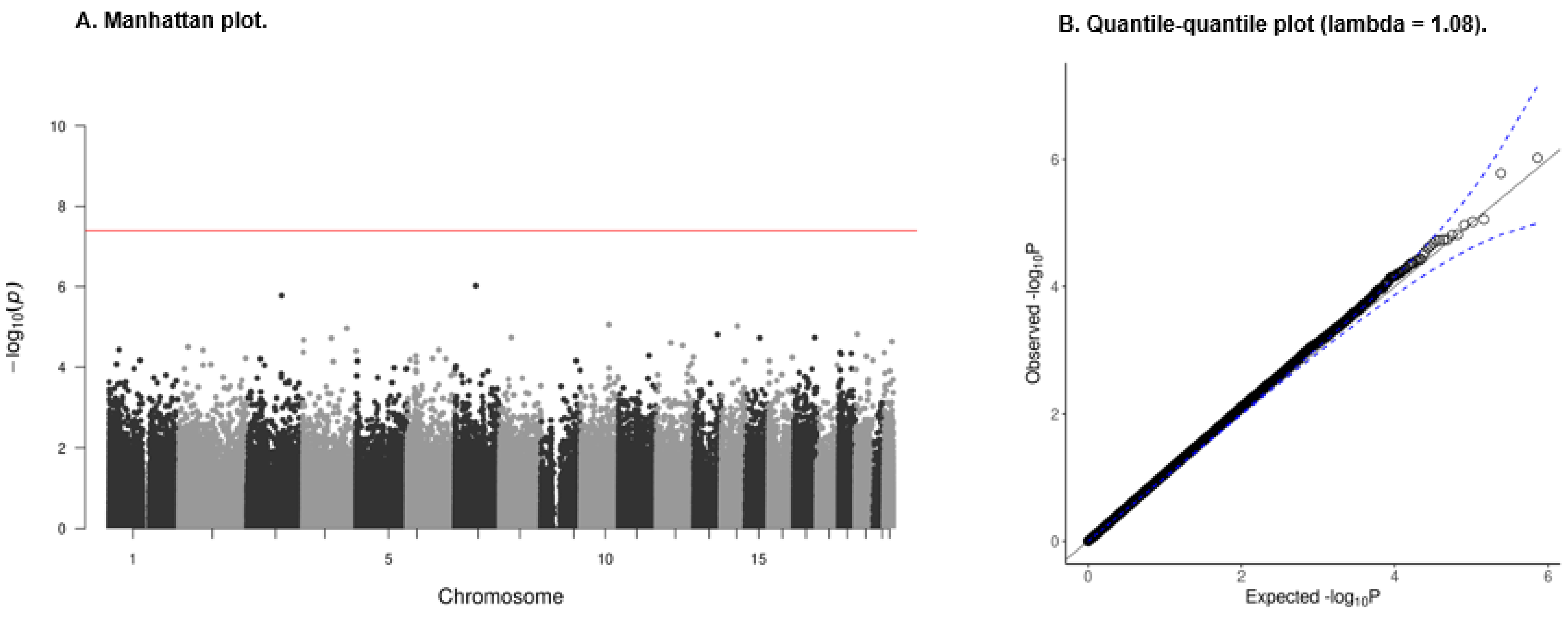

In panel A, the x axis shows the location of the CpG and the y axis shows the -log10(p) of the observed meta-analytical association between DNAm at this CpG at birth and parent-rated sleep fragmentation problems in childhood. The red line corresponds to the cut-off of statistical significance after multiple-testing correction ($p<4.0\times10^{-8}$). Panel B is a quantile-quantile plot that shows the distribution of observed p-values, compared to the distribution expected by chance.

[a] These plots depict associations between DNAm at *birth* and sleep fragmentation problems in childhood (n=1,681). In a sensitivity analysis, we re-ran the meta-analysis using results where DNAm in *childhood* was used to predict sleep fragmentation problems in childhood (n=1,156). The results remained unchanged: we observed no associations between DNAm and sleep fragmentation problems ($p<4.0\times10^{-8}$) (lambda=1.00).

Figure S4. DNAm and actigraphy-estimated child sleep duration among school-aged children: Manhattan and quantile-quantile plots. [a]

A. Manhattan plot.

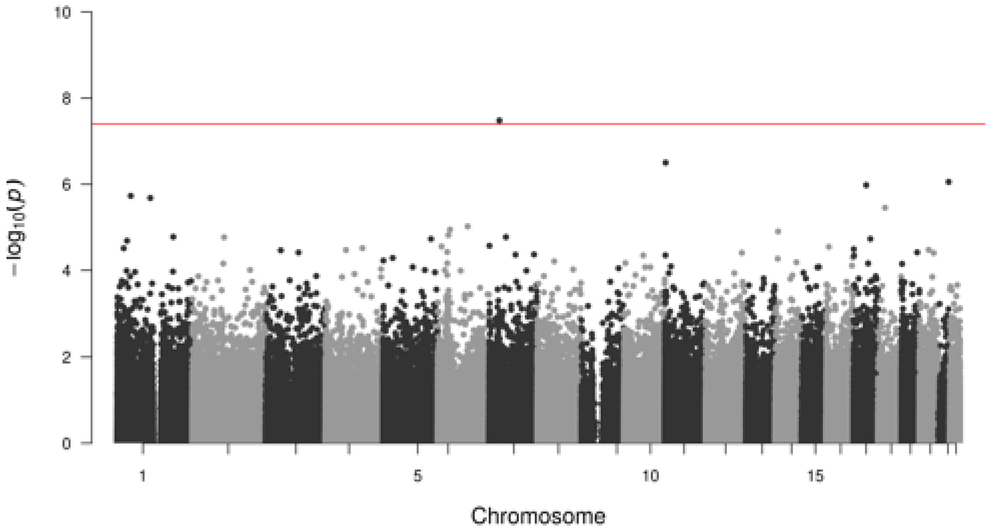

B. Quantile-quantile plot (lambda = 1.05).

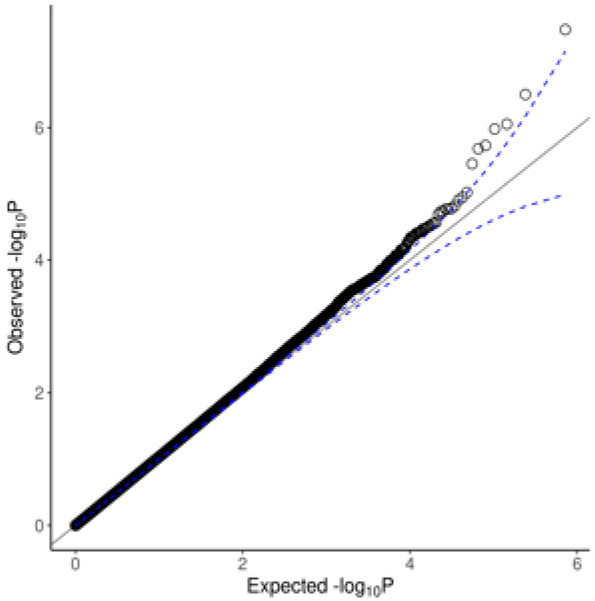

In panel A, the x axis shows the location of the CpG and the y axis shows the -log10(p) of the observed meta-analytical association between DNAm at this CpG at birth and actigraphy-estimated sleep duration in childhood. The red line corresponds to the cut-off of statistical significance after multiple-testing correction ($p<4.0\times10^{-8}$). Panel B is a quantile-quantile plot that shows the distribution of observed p-values, compared to the distribution expected by chance.

[a] These plots depict associations between DNAm at *birth* and sleep duration in childhood (n=582). In a sensitivity analysis, we re-ran the meta-analysis using results where DNAm in *childhood* was used to predict sleep duration in childhood (n=717). We observed no associations between DNAm and actigraphy-based sleep duration ($p<4.0\times10^{-8}$) (lambda=0.95).

Figure S5. DNAm and actigraphy-estimated child sleep-onset-latency among school-aged children: Manhattan and quantile-quantile plots. [a]

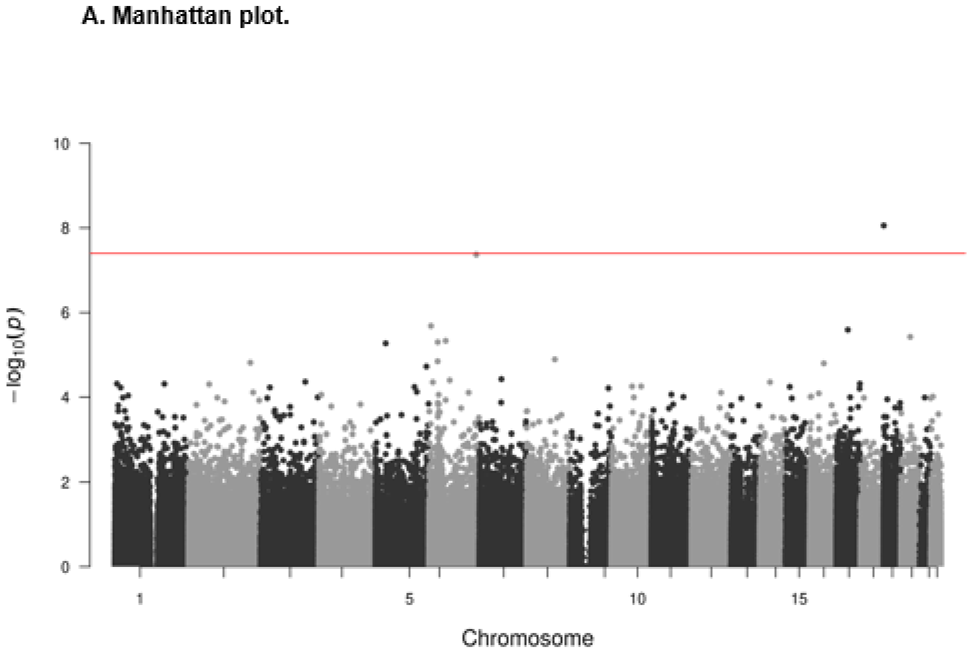

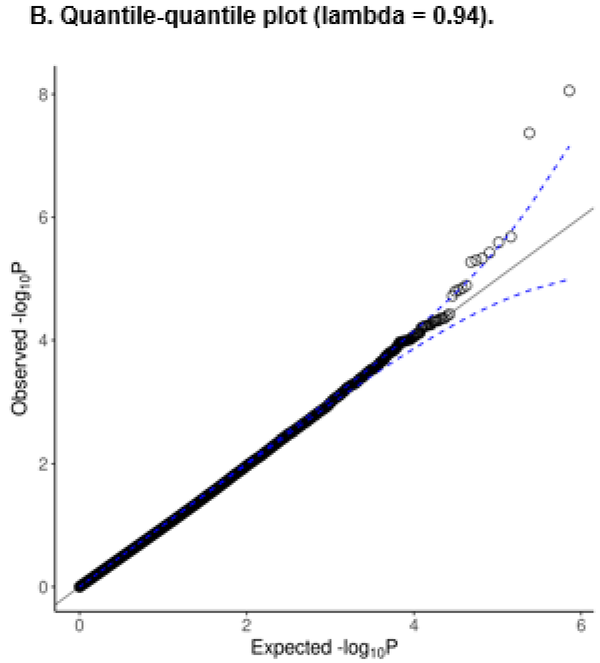

In panel A, the x axis shows the location of the CpG and the y axis shows the -log10(p) of the observed meta-analytical association between DNAm at this CpG at birth and actigraphy-estimated sleep-onset-latency in childhood. The red line corresponds to the cut-off of statistical significance after multiple-testing correction ($p<4.0\times10^{-8}$). Panel B is a quantile-quantile plot that shows the distribution of observed p-values, compared to the distribution expected by chance.

[a] These plots depict associations between DNAm at *birth* and sleep-onset-latency in childhood (n=582). In a sensitivity analysis, we re-ran the meta-analysis using results where DNAm in *childhood* was used to predict sleep-onset-latency in childhood (n=717). We observed no associations between DNAm and sleep initiation problems ($p<4.0\times10^{-8}$) (lambda=0.98).

Figure S6. DNAm and actigraphy-estimated child wake-after-sleep-onset duration among school-aged children: Manhattan and quantile-quantile plots. [a]

A. Manhattan plot.

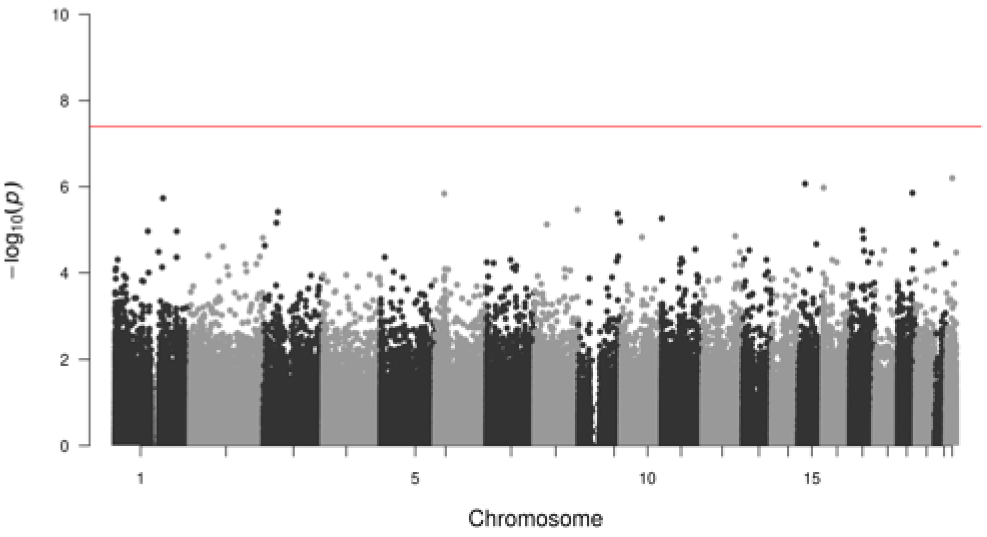

B. Quantile-quantile plot (lambda = 1.08).

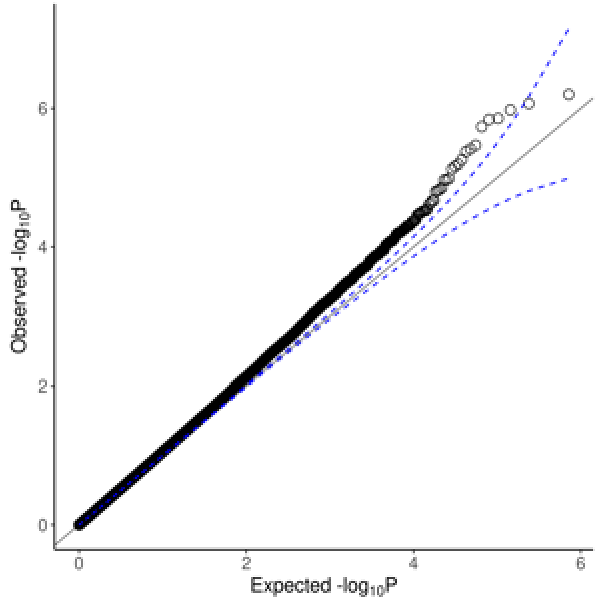

In panel A, the x axis shows the location of the CpG and the y axis shows the -log10(p) of the observed meta-analytical association between DNAm at this CpG at birth and actigraphy-estimated wake-after-sleep-onset duration in childhood. The red line corresponds to the cut-off of statistical significance after multiple-testing correction ($p<4.0\times10^{-8}$). Panel B is a quantile-quantile plot that shows the distribution of observed p-values, compared to the distribution expected by chance.

[a] These plots depict associations between DNAm at *birth* and wake-after-sleep-onset duration in childhood (n=582). In a sensitivity analysis, we re-ran the meta-analysis using results where DNAm in *childhood* was used to predict wake-after-sleep-onset duration problems in childhood (n=716). The results remained unchanged: we observed no associations between DNAm and wake-after-sleep-onset duration ($p<4.0\times10^{-8}$) (lambda=1.09).
