# Supplementary material for: Longitudinal associations of DNA methylation and sleep in children: a meta-analysis
Source: Clin Epigenetics. 2022 Jul 5;14:83. doi: 10.1186/s13148-022-01298-4 (PMC9258202; doi:10.1186/s13148-022-01298-4)
Supplement: Supplementary file 2 — Additional file2. Methods [file 13148_2022_1298_MOESM2_ESM.docx]

Longitudinal associations of DNA methylation and sleep in children: A meta-analysis

Supplementary Methods

**CONTENTS:**

[ALSPAC 2](#_Toc85026281)

[CHOP 4](#_Toc85026282)

[EDEN 7](#_Toc85026283)

[Generation R 10](#_Toc85026284)

[GLAKU 13](#_Toc85026285)

[Healthy Start 16](#_Toc85026286)

[HELIX 18](#_Toc85026287)

[INMA 21](#_Toc85026288)

[LINA 24](#_Toc85026289)

[MoBa-1 and MoBa-2 26](#_Toc85026290)

[PREDO 28](#_Toc85026291)

[PROGRESS 30](#_Toc85026292)

[Project Viva 33](#_Toc85026293)

[References 35](#_Toc85026294)

#

# ALSPAC

**Full name:** The Avon Longitudinal Study of Parents and Children

**Cohort profile:**Boyd et al. (2013),^1^Fraser et al. (2013)^2^

**Design and study population:** ALSPAC is a prospective cohort study based in the South West of England, UK. Pregnant women resident in Avon, UK with expected dates of delivery 1st April 1991 to 31st December 1992 were invited to take part in the study. The initial number of pregnancies enrolled is 14,541 (for these at least one questionnaire has been returned or a “Children in Focus” clinic had been attended by 19/07/99). When the oldest children were approximately 7 years of age, an attempt was made to bolster the initial sample with eligible cases who had failed to join the study originally. The total sample size after the age of seven is 15,454 pregnancies, resulting in 15,589 fetuses. Of these, 14,901 were alive at 1 year of age. Detailed information has been collected on these women, their partners and their offspring at regular intervals to the present date. Please note that the study website contains details of all the data that is available through a fully searchable data dictionary and variable search tool: <http://www.bristol.ac.uk/alspac/researchers/our-data/>

**Consent and ethical approval:** Written informed consent has been obtained for all ALSPAC participants. Ethical approval for the study was obtained from the ALSPAC Ethics and Law Committee and the Local Research Ethics Committees.

**Division into subcohorts**: None

**Sleep measurements:** Sleep was assessed using mother-reported questionnaires. At mean age of 11.7 years (range 11.7-13.2), sleep duration was assessed based on a series of questions about the time at which their child usually wakes up and goes to sleep on school days and at the weekend (in hours and minutes). A variable for the average hours of sleep per night was derived based on the formula ((5*weekday hours/night + 2*weekend hours/night). At mean age of 9.6 years (range 9.6-11.0), sleep initiation was assessed based on the question: “In the past year, has your child regularly had difficulty going to sleep?”. A binary yes/no variable was generated based on the questionnaire response. At mean age of 9.6 years (range 9.6-11.0), sleep fragmentation was assessed based on the question: “How often during the night does your child usually wake (number of times)?” A binary yes (≥ 1 time)/no (0 times) variable was generated based on the questionnaire response.

**DNA sample collection:** DNA was extracted from cord blood collected at birth, and from child blood samples collected through venipuncture at the mean age of 7.5 years (range 7.1-9.1 years).

**Methylation measurements:** As part of the ARIES (Accessible Resource for Integrated Epigenomic Studies, http://www.ariesepigenomics.org.uk/) project, the *Illumina Infinium® HumanMethylation450 BeadChip* has been used to generate epigenetic data on 1,018 mother-offspring pairs in the ALSPAC cohort. DNA methylation data for cord blood in the neonates and peripheral blood (whole blood, buffy coats or blood spots) in the children at age 7 were included in this analysis. DNA extraction, wet laboratory preparation and DNA methylation measurement were performed as part of the ARIES project, as described previously.^3,4^ Briefly, samples from all ARIES time-points were distributed semi-randomly across HM450 slides to minimize the potential for confounding by technical batch. Data pre-processing was performed using the *meffil* package.^4^ Samples failing quality control (average probe detection *p* value ≥ 0.01, those with sex or genotype mismatches) were excluded from further analysis, and probes containing < 95% of signals detectable above background signal (detection *p* value < 0.01) were also removed. Functional normalization was performed to minimize non-biological variation in probes.

**Cell type correction:** We used the Houseman method to adjust for cell composition, using the Bakulski cord blood reference panel for all cord blood samples.

**Batch correction:** Adjustment for batch effects was done by surrogate variables for technical batch, using the *sva* package in R.^5^

**Ancestry/ethnicity:** Analyses were conducted on participants of European ancestry only.

**Maternal education:** Maternal educational attainment reported during pregnancy was categorized into low (high school education or less) vs high (more than high school education).

**Smoking during pregnancy:** Maternal self-reported smoking during pregnancy was categorized into never, smoking in the first trimester and smoking which continued after the first trimester.

**Gestational age at birth:** Gestational age at birth was calculated based on the date of the mother’s last menstrual period or from ultrasound.

**Study funding:** The UK Medical Research Council and Wellcome (Grant ref: 217065/Z/19/Z) and the University of Bristol provide core support for ALSPAC. This publication is the work of the authors and RCR will serve as the guarantor for the contents of this paper. A comprehensive list of grants funding is available on the ALSPAC website (<http://www.bristol.ac.uk/alspac/external/documents/grant-acknowledgements.pdf>); This research was specifically funded by the BBSRC (BBI025751/1 and BB/I025263/1) as part of the ARIES project.

**Acknowledgements:** We are extremely grateful to all the families who took part in this study, the midwives for their help in recruiting them, and the whole ALSPAC team, which includes interviewers, computer and laboratory technicians, clerical workers, research scientists, volunteers, managers, receptionists and nurses.

# CHOP

**Full name:** European Childhood Obesity Project Study

**Cohort profile**: Koletzko et al (2009),^6^ Weber et al (2014),^7^ Rzehak et al (2016)^8^

**Design and study population**: The European Childhood Obesity Project (CHOP) is a randomized controlled intervention trial originally designed to assess the impact of formula composition (lower vs. higher protein content) on growth and development among healthy infants born between October 2002 and July 2004. Across five European countries (Germany, Belgium, Italy, Poland, Spain), 1678 neonates and their mothers were enrolled in the study within the first 8 weeks after delivery (mean 14 days). After the one year intervention, children were prospectively followed-up until the age of 11 years. The current study is based on a subset of 583 children who participated in the 11-year follow-up. Of these children, 444 had sleep data recorded, and 430 had whole blood methylation measured (11 failing quality control). A total of 328 participants had both valid sleep and methylation data. Of these individuals, 20 were missing information on maternal smoking and were excluded from all analyses, and 11 were missing the child psychopathology score and were excluded from analyses involving this score. Thus, the final sample included 308 children (108 from Spain, 85 from Italy, 53 from Germany, 42 from Belgium, and 20 from Poland).

**Consent and ethical approval**: Written informed consent has been obtained from all parents and children. Local ethics committees of each study center approved the trial: Belgium (Comité d’Ethique de L’Hopital Universitaire des Enfants Reine Fabiola; No. CEH 14/02), Germany (Bayerische Landesärztekammer Ethik-Kommission; No. 02070), Italy (Azienda Ospedaliera San Paolo Comitato Etico; No 14/2002), Poland (Instytut Pomnik-Centrum Zdrowia Dziecka Komitet Etyczny; No 243/KE/2001), and Spain (Comité ético de investigación clínica del Hospital Universitario de Tarragona Joan XXIII). The study was registered at clinicaltrials.gov as NCT00338689.

**Division into subcohorts**: None

**Sleep measurements**: Sleep was assessed using the SenseWear™ Armband 2 (SWA) (Body Media Inc., Pittsburgh, PA) at a mean age of 11.1 years (range 10.7 to 11.6 years). According to the study protocol, children were told to wear the SWA on at least 3 consecutive days for at least 20 hours per day. The device is worn on the right upper arm over the triceps muscle and collects data in 1 min epochs through five sensors. An inclinometer measures whether the user is standing or lying down (scored as “lying” and “not lying”). The SenseWear armband also gives sleep data (as calculated by an inherent algorithm) in 1 min epochs. The armband was shown to be a valid tool for sleep assessment analysis in children.^9,10^ However, the armband was less accurate at an individual level than the more commonly used actigraph.^11^ As no modification or further inspection of the sleep algorithm in the proprietary software is possible, we chose to process the data with an algorithm more similar to algorithms used with actigraph devices with scoring rules to identify sleep bouts by minutes without activity.^12^ To do so we combined the inclinometer information with the inherent SenseWear sleep variable. To avoid inclusion of daytime naps into the sleep data, we applied it only to timeframes from 6 pm to 9 am. No outliers were removed.

**DNA sample collection:** DNA was extracted from child blood samples collected through venipuncture at the mean age of 11.1 years (range 10.7 to 11.6 years).

**Methylation measurements**: We used the *Illumina Infinium® HumanMethylation450 BeadChip* to measure DNA methylation in child blood. DNA extraction, bisulfite conversion and methylation analysis were performed at the Genome Analysis Center of Helmholtz Zentrum München, Munich, Germany. Details were described previously.^8^ In brief, genomic DNA was extracted using a standard precipitation procedure. Bisulfite conversion was performed using the EZ-96 DNA Methylation Kit (Zymo Research, Irvine, Ca; USA) and converted DNA samples were hybridized on the Infinium HumanMethylation450 BeadChip (HM450K) according to the manufacturer's instructions (Illumina Inc., San Diego, USA).

467 samples were measured in the laboratory using the Illumina 450K chip. Of these, 24 were replicates of samples from participants at an earlier time point (age 5.5, T66), and 13 were technical replicates. Quality control and normalization were primarily based on the *CPACOR* method by Lehne et al,^13^ using R version 3.5.2., and *minfi* version 1.28.4.^14^ The sex of the participants was predicted using the *minfi* command *getSex*. For 9 samples, the predicted sex did not match the sex given by the participant and these samples were removed from analysis. Raw intensities were read in from *idat* files using the *read.metharray* command, and subjected to background correction. A detection p threshold was set at 0.01: probes with detection p values greater than 0.01 were set to missing. Samples with greater than 5% missing values were eliminated (2 participants). As per Lehne et al., probes' intensities were divided into six categories based on probe type and color channel: type II red, type II green, type I red unmethylated, type I red methylated, type I green unmethylated and type I green methylated. Each category was quantile normalized separately using the *limma* (v 3.38.3) command *normalizeQuantiles*. Probes from the autosomes were normalized separately to those from the sex chromosomes. No filtering of the probes was performed during these cohort-level analyses. For samples with two replicates, the replicate with the higher detection rate was chosen for analysis purposes.

**Cell type correction**: We used the Houseman algorithm to estimate cell composition in these child DNA samples through the *minfi estimateCellCounts* command.^15^

**Batch correction**: Adjustment for batch effects was done by surrogate variables for technical batch, using the *sva* package in R.^5^

**Ancestry/ethnicity**: There were 5 study centers in 5 different countries: we adjusted for study center as a covariate rather than performing stratified analysis (stratified analysis would have resulted in too small samples sizes).

**Maternal education**: Mothers self-reported their educational level at enrollment.

**Smoking during pregnancy**: Mothers self-reported smoking during pregnancy at enrollment. This was categorized into never vs quit in early pregnancy vs continued smoking after early pregnancy.

**Gestational age at birth**: NA (cord blood analyses were not carried out; thus, this covariate was not needed).

**Study funding**: The CHOP study has been carried out with partial financial support from the Commission of the European Community, specific RTD Programme "Quality of Life and Management of Living Resources", within the Fifth Framework Program (research grants no. QLRT-2001-00389 and QLK1-CT-200230582), the Sixth Framework Program (contract no. 007036), and Seventh Framework Programme (EarlyNutrition; grant agreement no. 289346), the EU H2020 project LIFECYCLE under grant no. 733206 and the European Research Council Advanced Grant META-GROWTH (ERC-2012-AdG – no.322605) and with financial support from Polish Ministry of Science and Higher Education (2571/7.PR/2012/2). This manuscript does not necessarily reflect the views of the Commission and in no way anticipates the future policy in this area. No funding bodies had any role in the study design, data collection and analysis.

**Acknowledgements**: The authors would particularly like to thank all the cohort participants for their generous collaboration. Furthermore, thanks to all persons who designed and conducted the study, entered the data, and participated in the data analysis and who are represented by the European Childhood Obesity Trial Study Group participants: B Koletzko, V Grote, M Totzauer, K Gürlich, P Schwarzfischer, N Aumüller, V Luque, M Zaragoza-Jordana, N Ferré, J Escribano, R Closa-Monasterolo, A Xhonneux, Jean-Paul Langhendries, E Verduci, E Riva, D Gruszfeld.

# EDEN

**Full name:** EDEN Mother-Child Cohort

**Cohort profile**: Heude et al (2016)^16^

**Design and study population**: The EDEN (*Etude des Déterminants pré et post natals du développement et de la santé de l′Enfant*) Mother-Child Cohort is a longitudinal cohort study was set up in 2003 in two university maternity clinics, in Nancy and Poitiers, France. Pregnant women seen for a prenatal visit at the departments of Obstetrics and Gynecology of the University Hospital of Nancy and Poitiers before their twenty-fourth week of amenorrhea were invited to participate. Enrolment started in February 2003 in Poitiers and September 2003 in Nancy; it lasted 27 months in each center. Among eligible women, 55% (n=2002) accepted to participate. The children’s health and behavior was regularly followed-up for up to 8 years from birth, by visits to research centers and questionnaires mailed to parents. Exclusion criteria were multiple pregnancies, known diabetes before pregnancy, French illiteracy or planning to move out of the region within the next 3 years. More information on EDEN can be found at its website (<https://eden.vjf.inserm.fr/index.php?lang=en> ).

**Consent and ethical approval**: All participants provided informed consent. The study received approval from the ethics committee (CCPPRB) of Kremlin Bicêtre on 12 December 2002 and from CNIL (*Commission Nationale Informatique et Liberté*), the French data privacy institution.

**Division into subcohorts**: None

**Sleep measurements**: Sleep was assessed using parent-rated questionnaires at mean age of 5.7 years (range 5.0 to 6.1 years). A parental questionnaire on child’s health and behaviors was administered at the same age, which included questions on the children's sleeping habits. The sleep duration time was calculated as the difference (in hours) between the time at waking up in the morning and the time at going to bed in the evening, as reported by parents. The sleep duration time was standardized using z-score, and outlier scores were winsorized beyond 3SD. Data on difficulties in sleep initiation and sleep fragmentation were obtained using the following questions: “during the last month, when you put your child in bed in the evening, does he have difficulties in falling asleep?” and “has your child woken up during the night in the last month?”. The variables on sleep initiation and sleep fragmentation were dichotomized as follows: “no” if the answer was “never or nearly never”, or “yes” if the answer was “sometimes”, “one night every two”, “frequently”, “every evening or nearly every evening”.

**DNA sample collection:** DNA was extracted from cord blood collected at birth, and from child blood samples collected through venipuncture at the mean age of 5.7 years (range 5.0 to 6.1 years).

**Methylation measurements**: We used the *Illumina Infinium® HumanMethylation450 BeadChip* to measure DNA methylation in cord blood and child blood. DNA was extracted from 162 cord blood samples using the QIAamp blood kit (Qiagen or equivalent protocols), followed by precipitation-based concentration using GlycoBlue (Ambion). DNA concentration was determined by Nanodrop measurement and Picogreen quantification. 500 ng of DNA was bisulphite-converted using the EZ 96-DNA methylation kit (Zymo Research), following the manufacturer’s standard protocol. After verification of the bisulphite conversion step using Sanger Sequencing, genome-wide DNA methylation was measured using the Illumina Infinium HumanMethylation450 BeadChip. After normalization of the concentration, the samples were randomized to avoid batch effects, and all paired samples were hybridized on the same chip. In total, 439,306 CpGs are available in children with DNA measurements.

**Cell type correction:** We used the Houseman method^15^ to estimate child blood cell composition using the *estimateCellCounts* function in the *minfi* R package.^14^ For the cord blood samples, the same approach but with the Bakulski cord blood reference panel was used.^15,17^

**Batch correction:** Adjustment for batch effects was done by surrogate variables for technical batch, using the *sva* package in R.^5^

**Ancestry/ethnicity**: Ancestry was not included as covariate in the models, as EDEN methylation data were collected only on Caucasian children born to French-speaking mothers.

**Maternal education**: Self-reported education during pregnancy was classified into “low” if completed primary or lower secondary education (ISCED level 0-2); or “medium/high” if completed upper secondary education or tertiary education (ISCED level 3-8).

**Smoking during pregnancy**: Mothers self-reported smoking was assessed using a questionnaire during mid-pregnancy and was categorized into never vs quit in early pregnancy vs continued smoking after early pregnancy.

**Gestational age at birth:** Gestational age at birth was calculated based on the date of the mother’s last menstrual period or from ultrasound.

**Study funding**: Foundation for Medical Research (FRM), National Agency for Research (ANR), National Institute for Research in Public Health (IRESP: TGIR cohorte santé 2008 program), French Ministry of Health (DGS), French Ministry of Research, Inserm Bone and Joint Diseases National Research (PRO-A) and Human Nutrition National Research Programs, Paris–Sud University, Nestlé, French National Institute for Population Health Surveillance (InVS), French National Institute for Health Education (INPES), the European Union FP7 programmes (FP7/2007-2013, HELIX, ESCAPE, ENRIECO, MEDall projects), Diabetes National Research Program (through a collaboration with the French Association of Diabetic Patients (AFD)), French Agency for Environmental Health Safety (now ANSES), MutuelleGénérale de l’EducationNationale (MGEN), French National Agency for Food Security, Health and Environment-wide Associations based on Large population Surveys (HEALS) and the French-speaking association for the study of diabetes and metabolism (ALFEDIAM). The funders had no role in study design, data collection and analysis, decision to publish, or preparation of the manuscript.

**Acknowledgements**: We are indebted to all the children and their parents for participation, as well as to the research nurses, research assistants, and laboratory personnel involved in the EDEN study. WE are also greatful to the the EDEN Mother–Child Cohort Study Group which includes: I. Annesi-Maesano, J.Y Bernard, J. Botton, M.A. Charles, P. Dargent-Molina, B. de Lauzon-Guillain, P. Ducimetière, M. de Agostini, B. Foliguet, A. Forhan, X. Fritel, A. Germa, V. Goua, R. Hankard, B. Heude, M. Kaminski, B. Larroque†, N. Lelong, J. Lepeule, G. Magnin, L. Marchand, C. Nabet, F. Pierre, R. Slama, M.J. Saurel-Cubizolles, M. Schweitzer, O. Thiebaugeorges.

# Generation R

**Full name:** The Generation R Study

**Cohort profile**: Kooijman et al (2016)^18^

**Design and study population**: The Generation R Study is a population-based prospective cohort study. All pregnant women living in Rotterdam, the Netherlands, with an expected delivery date between April 2002 and January 2006 were invited to participate. These women and their children have been followed at regular intervals since recruitment. For the current study, only participants who reported being of Dutch ethnicity were included.

**Consent and ethical approval**: Written informed consent was obtained for all participants. The Generation R Study is conducted in accordance with the World Medical Association Declaration of Helsinki and was approved by the Medical Ethical Committee of the Erasmus MC, University Medical Center Rotterdam.

**Division into subcohorts**: None

**Sleep measurements**: Sleep duration was assessed using parent-rated questionnaires at the mean age of 9.69 years (range 9.01-11.59). Parents were asked about the mean sleep duration of their child using the question: “How many hours of sleep does your child get on most nights?”. For sleep initiation problems, we used the Child Behavior Checklist question, ‘Trouble sleeping’. This questionnaire was completed by parents at mean age of 11,69 years (range 10.57-11.97).

At the mean age of 11.69 years (range 10.57-11.97), sleep duration, sleep onset latency and waking after sleep onset were estimated with wrist tri-axial actigraphy (GENEActiv; Activinsights, UK) worn on the non-dominant wrist for nine subsequent days (five school days and four weekend days).^19^ Each morning children filled out sleep diaries answering questions about their sleep timing (e.g. the time they went to bed). This was used as input to guide actigraphy analyses. The binary files were processed using the R-package *GGIR*.^20^

Originally, parents also responded to the question "The child wakes up more than twice per night", which could be used to identify sleep fragmentation problems. However, due to issues arising from the small number of participants that were classified as having caregiver-reported sleep fragmentation problems and DNAm data at birth (n=16) or in childhood (n=12), these models were dropped during cohort-level quality control and are not included in the meta-analysis. Nonetheless, as Generation R was one of only two cohorts that had data on all six phenotypes of interest, we used sleep fragmentation data to describe the independence of the different phenotypes.

**DNA sample collection:** DNA was extracted from cord blood collected at birth, and from child blood samples collected through venipuncture at the mean age of 9.76 years (range 8.58-11.18 years).

**Methylation measurements**: We used the *Illumina Infinium® HumanMethylation450 BeadChip* to measure DNA methylation in cord blood and in child blood. Preparation and normalization of the DNA methylation data was performed according to the *CPACOR* workflow^13^ using the software package *R* (The R Core Team, 2013). In detail, the *idat* files were read using the *minfi* package.^14^ Probes that had a detection p-value above background (based on sum of methylated and unmethylated intensity values) greater to or equal to 1E-16 were set to missing per array. Next, the intensity values were stratified by autosomal and non-autosomal probes and quantile normalized for each of the six probe type categories separately: type II red/green, type I methylated red/green and type I unmethylated red/green. Beta values were calculated as proportion of methylated intensity value on the sum of methylated+unmethylated+100 intensities. Arrays with observed technical problems such as failed bisulfite conversion, hybridization or extension, as well as arrays with a mismatch between sex of the proband and sex determined by the chromosome X and Y probe intensities were removed from subsequent analyses. Additionally, only arrays with a call rate >95% per sample were processed further. Probes on the X and Y chromosomes were excluded from analyses. The final dataset contained information on 469,242 CpGs for 1,396 samples at birth.

**Cell type correction**: We used the Houseman algorithm to estimate cell composition.^15^ In the cord blood DNAm analysis, the Bakulski cord blood reference panel^17^ was used to estimate cell composition and in the childhood DNAm analyses, the Reinius reference panel^21^ was used.

**Batch correction**: Adjustment for batch effects was done by surrogate variables for technical batch, using the *sva* package in R.^5^

**Ancestry/ethnicity**: For the current study, only mothers of European ancestry were included: due to the ethnic homogeneity of the sample, ancestry was not included as a covariate.

**Maternal education**: Maternal education was defined by the highest attained educational level and classified into two categories (low and medium/high education).

**Smoking during pregnancy**: Smoking during pregnancy was assessed with postal questionnaires in early pregnancy (gestational age <18 weeks), mid pregnancy (gestational age 18-25 weeks) and late pregnancy (gestational age>25 weeks). It was classified in three categories: “Never smoked during pregnancy”, “Quit when pregnancy was known” and “Continued during pregnancy”. In analyses we used two dummy variables with “Never smoked during pregnancy” as the reference category.

**Gestational age at birth**: Gestational age at delivery came from medical records.

**Study funding**: The general design of the Generation R Study is made possible by financial support from Erasmus MC, Erasmus University Rotterdam, the Netherlands Organization for Health Research and Development and the Ministry of Health, Welfare and Sport. The EWAS data were funded by a grant from the Netherlands Genomics Initiative (NGI)/Netherlands Organisation for Scientific Research (NWO) Netherlands Consortium for Healthy Aging (NCHA; project nr. 050‐060‐810), by funds from the Genetic Laboratory of the Department of Internal Medicine, Erasmus MC, and by a grant from the National Institute of Child and Human Development (R01HD068437). This study received support from the Erasmus Medical Center Efficiency Grant (MRC‐2013‐169) and a grant of the Dutch Ministry of Education, Culture, and Science and the Netherlands Organization for Scientific Research (NWO grant No. 024.001.003, Consortium on Individual Development) to Dr Tiemeier, and Dr Tiemeier was supported by a grant from NWO (016.VICI.170.200). The work of Dr Sammallahti was funded by the ‘LEaDing Fellows’ EU Marie Skłodowska-Curie COFUND Programme. This project has also received funding from the European Union’s Horizon 2020 Research And Innovation Programme under the grant agreement numbers 733206 (LifeCycle), [848158](https://www.sciencedirect.com/science/article/pii/S0149763419307791#gs0005) (EarlyCause), 874739 (LongITools), 824989 (EUCAN-Connect), and under the Marie Skłodowska‐Curie grant agreement number No 707404 awarded to Dr Cecil, and additionally from the European Joint Programming Initiative ‘A Healthy Diet for a Healthy Life’ (JPI HDHL, NutriPROGRAM project, ZonMw the Netherlands no. 529051022). The authors have declared that they have no competing or potential conflicts of interest.

**Acknowledgements**: The authors thank all participants and parents, students, practitioners, hospitals, midwives, and pharmacies. The Generation R Study is conducted by Erasmus MC, University Medical Center Rotterdam in close collaboration with the School of Law and Faculty of Social Sciences of the Erasmus University Rotterdam, the Municipal Health Service Rotterdam area, Rotterdam, the Rotterdam Homecare Foundation, Rotterdam and the *Stichting Trombosedienst & Artsenlaboratorium Rijnmond* (STAR‐MDC), Rotterdam. The generation and management of the Illumina 450K methylation array data (EWAS data) for the Generation R Study was executed by the Human Genotyping Facility of the Genetic Laboratory of the Department of Internal Medicine, Erasmus MC, and the Netherlands. The authors thank all colleagues involved in generation and management of methylation data and genotyping.

# GLAKU

**Full name:** Glycyrrhizin in Licorice Study

**Cohort profile**: Strandberg et al (2001)^22^

**Design and study population**: The adolescents of the GLAKU (Glycyrrhizin in Licorice) cohort came from an urban community-based cohort comprising 1049 infants born between March and November 1998 in Helsinki, Finland.^22^ In 2009–2011, initial cohort members who had given permission to be contacted and whose addresses were traceable (n=920, 87.7% of the original cohort in 1998) were invited to a follow-up, of which 692 (75.2%) could be contacted by phone (mothers of the adolescents). Of them, 451 (65.2% of those who could be contacted by phone, 49% of the invited) participated in a 12-year-follow-up.

**Consent and ethical approval**: Informed consent was obtained from all participants. The study protocol was approved by the ethical committees of the City of Helsinki and the Uusimaa Hospital District.

**Division into subcohorts**: None

**Sleep measurements**: The caregiver-reported sleep was assessed with questionnaire at mean age of 12.4 years (range 11.1-13.0 years). The sleep duration was assessed by question “How many hours of sleep does your child get on most nights” with five categories: 9-11 hours (coded as 10 hours), 8-9 hours (coded as 8.5 hours), 7-8 hours (coded as 7.5 hours), 5-7 hours (not in data) or less than 5 hours (not in data). Sleep duration was treated as continuous and transformed into a z-score. Difficulties initiating sleep was assessed by item “The child has difficulty getting to sleep at night” and the sleep fragmentation was assessed by item “The child wakes up more than twice per night”. Both items were assessed in a five-category scale: never, occasionally, sometimes, often or always. Difficulties initiating sleep was dichotomized as never/occasionally vs sometimes/often/always. Sleep fragmentation was dichotomized never vs occasionally/sometimes/often/always because in this item only a few participants

had selected sometimes, often or always.

Sleep was assessed using actigraphy at mean age of 12.4 years (range 11.1-13.0 years). The details have been reported previously.^23^ The Actiwatch AW7 (Cambridge Neurotechnology Ltd., UK) were worn on the non-dominant wrist. Both weekdays and weekends were included in the sleep measurements in order for the measurement period to represent typical sleep over an average of 8 nights. Adolescents were instructed to mark the wake-up time and bedtime by pressing a button in the device and by completing a sleep log, and register any temporary pauses into the sleep log. Nights were excluded from further sleep analyses if the actigraph was not in use, information on bedtimes was missing, the data on reported bedtime indicated the child was already asleep (suggesting the bedtime was not correctly reported), in­formation on waking time was missing and the activity pattern was unclear, or the parent reported a change in normal life due to, for example, illness or travel. Sleep time, sleep onset latency and wake after sleep onset were determined by Actiwatch Activity & Sleep Analysis version 7.38 software (Cambridge Neurotechnology, UK).

**DNA sample collection:** DNA was extracted from child blood samples collected through venipuncture at the mean age of 12.4 years (range 11.1-13.0 years).

**Methylation measurements**: We used the *Illumina Infinium® HumanMethylationEPIC BeadChip* to measure DNA methylation in child blood. DNA was extracted at the National Institute for Health and Welfare, Helsinki, Finland and the Department of Medical and Clinical Genetics, University of Helsinki, Finland [LJMT1] and methylation analyses were performed at the Max Planck Institute in Munich, Germany. DNA was bisulphite-converted using the EZ-96 DNA Methylation kit (Zymo Research). Genome-wide methylation status of over 850 000 CpG sites was measured using the Infinium Methylation EPIC array (Illumina Inc., San Diego, USA) according to the standard protocol in 240 blood samples. The arrays were scanned using the iScan System (Illumina Inc., San Diego, USA). The quality control pipeline was set up using the R-package *minfi*. Methylation beta-values were normalized using the *funnorm* function. One ID showed density artefacts after normalization and was removed from further analysis. We excluded any probes on chromosome X or Y, probes containing SNPs and cross-hybridizing probes according to Chen et al (2013),^24^ Price et al (2013),^25^ and McCartney et al (2016).^26^ Furthermore, any CpGs with a detection p-value > 0.01 in at least 25% of the samples were excluded. The final dataset contains 812,943 CpGs and 239 IDs.

**Cell type correction**: Cell type proportions in child blood samples were estimated using the *estimateCellCounts* function in the *minfi* R package,^14^ based on the method developed by Houseman and colleagues,^15^ using the Reinius reference panel^21^ to estimate the proportions of six white blood cell subtypes (CD4+ T-lymphocytes, CD8+ T-lymphocytes, NK (natural killer) cells, B-lymphocytes, monocytes and granulocytes).

**Batch correction**: We used *ComBat* to check and adjust for the batch effects.

**Ancestry/ethnicity**: We used GWAS-based principal component data to adjust for ancestry. Genotyping was performed on Illumina Human OmniExpress Exome 1.2 bead chip (Illumina Inc., San Diego, CA) at the Tartu University, Estonia in September 2014 according to the standard protocols. Genomic coverage was extended by imputation using the 1000 Genomes Phase I integrated variant set (v3/April 2012; NCBI build 37/hg19) as the reference sample and IMPUTE2 software. Before imputing the following QC, filters were applied: SNP clustering probability for each genotype > 95%, Call rate > 95% individuals and markers (99% for markers with MAF < 5%), MAF > 1%, HWE p > 1*10–6. Moreover, heterozygosity, sex check, and relatedness checks were performed, and any discrepancies were removed (N = 2). We performed multi-dimensional scaling (MDS) analysis on the identity by state matrix of quality-controlled genotypes. The first three components depicted the origin admixture and were included as covariates in the regression analyses.

**Maternal education**: The maternal education was assessed by question “What is the highest education level you have achieved” with 8 categories: 1=four-to- eight-year primary school in the former Finnish school system, 2=six-year primary school in the current Finnish school system, 3=secondary education, 4=post-secondary degree, 5=bachelor’s degree, 6=master’s degree, 7=doctoral dissertation or 8=some other education. There were no occurrences of the categories 1 nor 8 in the data. For the analysis, the categories 2-3 were assigned to 0 and categories 4-7 to 1. This resulted in 35 individuals with the value 0 and 167 individuals with the value 1.

**Smoking during pregnancy**: Mothers self-reported whether they had smoked during pregnancy (yes/no)

**Gestational age at birth**: Based on ultrasound scans.

**Study funding**: The study has been supported by Academy of Finland, University of Helsinki, Hope and Optimism Initiative, Finnish Foundation for Pediatric Research, Sigrid Juselius Foundation, Jalmari and Rauha Ahokas Foundation, Signe and Ane Gyllenberg Foundation, Yrjo Jahnsson Foundation, Juho Vainio Foundation, Emil Aaltonen Foundation, and Ministry of Education and Culture, Finland. The 352 samples were genotyped at the Genotyping and Sequencing Core Facility of the Estonian Genome Centre, University of Tartu.

**Acknowledgements**: We thank all the GLAKU children and their parents for their enthusiastic participation. We also thank all the research nurses, research assistants, and laboratory personnel involved in the GLAKU study.

# Healthy Start

**Full name:** Healthy Start

**Cohort profile**: None published yet.

**Design and study population**: Healthy Start is a prospective pre-birth cohort study that enrolled 1,410 pregnant women in Colorado, USA in 2009-2014. Women were excluded if they had a multiple gestation, were younger than 16 years old, were beyond 24 weeks of completed gestation at enrollment, had a previous stillbirth or extremely preterm birth, or had serious chronic medical conditions. For the current study, we included only children aged 4 years and older at the time of the parent-completed sleep questionnaire, with Illumina Infinium HumanMethylation450 array data in cord blood. Of 362 meeting these criteria, we excluded 49 due to missing data on maternal smoking during pregnancy, 6 randomly selected siblings per each sibling pair, 16 who reported race/ethnicity other than the 3 primary groups, and finally 4 due to mismatch between reported and predicted sex (as described below under methylation measurements). Total sample size was therefore 283.

**Consent and ethical approval**: The Healthy Start study protocol was approved by the Colorado Multiple Institutional Review Board. All participants provided written informed consent.

**Division into subcohorts**: None

**Sleep measurements**: Sleep was assessed using a parent-rated questionnaire at mean age of 4.7 years (range 4.0 to 8.1 years). Parents completed the Sleep Disturbances Scale for Children, a 26-question survey with 5 response options per question. Sleep duration in response to the question, “How many hours of sleep does your child get on most nights?” was reported as 9-11 hours (coded as 10 hours), 8-9 hours (coded as 8.5 hours), 7-8 hours (coded as 7.5 hours), 5-7 hours (coded as 6 hours), or less than 5 hours (coded as 2.5 hours). This variable was treated as continuous, and transformed into a z-score. Values beyond 3*SD were winsorized. Sleep initiation was classified as a binary variable corresponding to an answer of never/occasionally vs sometimes/often/always, in response to the question, “The child has difficulty getting to sleep at night.” Sleep fragmentation was classified as a binary variable corresponding to an answer of never/occasionally vs sometimes/often/always, in response to the question, “The child wakes up more than twice per night.”

**DNA sample collection:** DNA was extracted from cord blood collected at birth.

**Methylation measurements**: We used the *Illumina Infinium® HumanMethylation450 BeadChip* to measure DNA methylation in cord blood. We removed 587 probes with high detection P value (>0.05). We removed 664 probes with a beadcount <3 in at least 5% of samples. We compared the predicted sex to the reported sex. Any samples with inconsistent sex were removed (n=4). The *preprocessQuantile* function in *minfi*^14^ was used to normalize. Outliers were removed using the 3*IQR method.

**Cell type correction**: Cell type proportions were estimated using *minfi* function *estimateCellCounts* with the Bakulski reference panel for cord blood.

**Batch correction**: *ComBat* was used for batch adjustment. Surrogate variables for technical batch were generated using *SVA*.

**Ancestry/ethnicity**: We included self-reported race/ethnicity, categorized into Non-Hispanic white (n=156), Hispanic (n=75), and non-Hispanic African-American (n=52), in the analytical models.

**Maternal education**: Maternal self-reported education during pregnancy was categorized into low (high school education or less) vs high (more than high school education).

**Smoking during pregnancy**: Maternal self-reported smoking during pregnancy was categorized into never, vs quit in early pregnancy, vs continued after early pregnancy.

**Gestational age at birth**: Gestational age at birth was recorded from medical records.

**Study funding**: The Healthy Start study was supported by grants from the National Institute of Diabetes and Digestive and Kidney Diseases (R01DK076648), the National Institute of Environmental Health Sciences (R01ES022934), and the Office of the Director (UH3OD023248) of the National Institutes of Health. APS was additionally supported by a grant from the National Institute of Environmental Health Sciences (R00ES025817).

**Acknowledgements**: None

# HELIX

**Full name:** Human Early Life Exposome

**Cohort profile**: Maitre et al (2018)^27^

**Design and study population**: Human Early Life Exposome (HELIX) study represents a collaborative project across six established and ongoing longitudinal population-based birth cohort studies in Europe, including the Born in Bradford (BiB) study in the UK, the Étude des Déterminants pré et postnatals du développement et de la santé de l’Enfant (EDEN) study in France, the INfancia y Medio Ambiente (INMA) cohort in Spain, the Kaunus cohort (KANC) in Lithuania, the RHEA Mother Child Cohort study in Crete, Greece, and the Norwegian Mother and Child Cohort Study (MoBa). HELIX had data available on parent-reported sleep duration and DNAm in childhood for five of the participating cohorts (BIB, EDEN, INMA-Sabadell, KAUNAS, and RHEA), and ran individual-participant pooled EWAS on these data. The HELIX project aims to measure and describe multiple environmental exposures from the different exposome domains during early life (pregnancy and childhood) and associate these with omics markers and child health outcomes.^27,28^

Note that INMA also had data on DNAm at birth and parent-reported sleep duration, initiation and fragmentation problems and actigraphy-estimated sleep duration, sleep-onset-latency and wake-after-sleep-onset duration; EDEN also had data on DNAm at birth and parent-reported sleep duration, initiation and fragmentation problems; and MoBa had data on DNAm at birth and parent-reported sleep duration. For these analyses, pooled HELIX data were not available: cohorts with relevant data participated as separate cohorts, as described in more detail under the headings “EDEN”, “INMA”, “MoBa-1 and MoBa-2”.

**Consent and ethical approval**: All six HELIX cohorts have the required permissions by national ethics committees for their cohort recruitment and follow-up visits and for secondary use of pre-existing samples and data. The work in HELIX was covered by new ethics approvals in each country. At enrolment in the HELIX project, families were asked to sign an informed consent form for the specific HELIX work including clinical examination and biospecimen collection and analysis. An Ethics Task Force was established to support the HELIX project on ethical issues, for advice on the project’s ethical compliance, identification and alerting to changes in legislation where applicable. Specific procedures are in place within HELIX to safeguard the privacy of study subjects and confidentiality of data.

**Division into subcohorts**: The Helix study cohort included participants from BIB, EDEN, INMA-Sabadell, KAUNAS, and RHEA cohorts. Data from these participating cohorts were pooled for the analysis. See “Design and study population”.

**Sleep measurements**: Sleep was assessed using mother-reported questionnaires. At a mean age of 7.7 years (range 5.4-12.0), sleep duration was assessed based on a series of questions about the earliest and latest time at which their child usually wakes up and goes to sleep on school days and at the weekend (in hours and minutes). A variable for the average hours of sleep per night was derived based on the formula ((5*weekday hours/night + 2*weekend hours/night).

**DNA sample collection:** DNA was obtained from buffy coat collected in EDTA tubes at age 7-9y. Briefly, DNA was extracted using the Chemagen kit (Perkin Elmer) in batches of 12 samples. Samples were extracted by cohort. DNA concentration was determined in a NanoDrop 1000 UV-Vis Spectrophotometer (ThermoScientific) and with Quant-iT™ PicoGreen® dsDNA Assay Kit (Life Technologies).

**Methylation measurements:** DNA methylation was assessed with the Infinium HumanMethylation450 beadchip from Illumina, following manufacturer’s protocol. Briefly, 700 ng of DNA were bisulfite converted using the EZ 96-DNA methylation kit following the manufacturer’s standard protocol, and DNA methylation measured using the Infinium protocol. A HapMap sample was included in each plate. In addition, 24 HELIX inter-plate duplicates were included. Samples were randomized taking into account cohort, sex and panel. Samples from the panel study (same subject) were processed in the same array. Two samples were repeated due to their overall low quality.

DNA methylation data was pre-processed using the *minfi* package.^14^ We increased the stringency of the detection p-value threshold to <10E-16, and probes not reaching a 98% call rate were excluded.^13^ Two samples were filtered due to overall low quality: one had a call rate <98% and the other did not pass QC parameters of the *MethylAid* package.^29^ Then, data was normalized with the functional normalization method, which also includes Noob background subtraction and dye-bias correction.^30^ After that, several quality control checks were performed. First, we checked sex consistency using the *shinyMethyl* package and excluded two samples.^31^ Genetic consistency of technical duplicates and samples from the same participant was checked with the 450k genotypes. In addition, genetic consistency was evaluated in those samples that had GWAS data and two of them were excluded. Principal component analysis showed no differential clusters, however a degree of grouping within the cluster was observed for cell type proportions, sex and cohort and for some technical variables. Slide batch effect was corrected with the *ComBat* method, see below.^32^ Finally, duplicated samples and HapMap samples were removed as well as control probes, probes designed to detect SNPs and probes to measure methylation levels at non-CpG sites.

**Cell type correction**: Cell type proportions were estimated using the estimateCellCounts function in the *minfi* R package^14^ based on the method developed by Houseman, and the Reinius reference panel.^15,21^ This estimated the proportion of B-cells, CD8 T-cells, CD4 T-cells, granulocytes, NK-cells and monocytes in each sample.

**Batch correction**: We used *ComBat* algorithm^32^ to adjust for potential batch effects, using slide as the major known technical bias.

**Ancestry/ethnicity**: All participants were of European ancestry based on self-reported information. Thus, ethnicity was not included as a covariate.

**Maternal education**: Mother self-reported their education level at intake. We categorized maternal education on high (University level or higher), medium (secondary education), or low (primary education, lower or no education)

**Smoking during pregnancy**: Maternal self-reported smoking status during pregnancy was classified into never smoked during pregnancy vs smoked during pregnancy.

**Gestational age at birth**: Gestational age at birth was estimated by 1) date of last menstrual period, 2) by ultrasound measurements or 3) registered in medical records, depending on the information available.

**Study funding**: The study has received funding from the European Community’s Seventh Framework Programme (FP7/2007-206) under grant agreement no 308333—the HELIX project. INMA data collections were supported by grants from the Instituto de Salud Carlos III, CIBERESP, and the Generalitat de Catalunya-CIRIT. KANC was funded by the grant of the Lithuanian Agency for Science Innovation and Technology (6-04-2014_31V-66). The Norwegian Mother and Child Cohort Study (MoBa) is supported by the Norwegian Ministry of Health and the Ministry of Education and Research, NIH/NIEHS (contract no. N01-ES-75558), and NIH/NINDS (grant no. 1 UO1 NS 047537-01 and grant no. 2 UO1 NS 047537-06A1). The Rhea project was financially supported by European projects, and the Greek Ministry of Health (Program of Prevention of Obesity and Neurodevelopmental Disorders in Preschool Children, in Heraklion district, Crete, Greece: 2011–2014; 'Rhea Plus': Primary Prevention Program of Environmental Risk Factors for Reproductive Health, and Child Health: 2012–2015). The work was also supported by MICINN (MTM2015-68140-R) and Centro Nacional de Genotipado-CEGEN-PRB2-ISCIII.

**Acknowledgements**: We would like to thank all the children and their families for their generous contribution.

# INMA

**Full name:** Infancia y Medio Ambiente

**Cohort profile**: Guxens et al (2012)^33^

**Design and study population**: The INMA—INfancia y Medio Ambiente—(Environment and Childhood) Project is a network of birth cohorts in Spain that aim to study the role of environmental pollutants in air, water and diet during pregnancy and early childhood in relation to child growth and development (<http://www.proyectoinma.org/>).

**Consent and ethical approval**: Written informed consent was obtained from all participating parents. The study was approved by the Ethical Committee of the Municipal Institute of Medical Investigation and by the Ethical Committee of the hospitals involved in the study.

**Division into subcohorts**: Data used for the current study comes from Sabadell subcohort of INMA. As all data came from the same subcohort, no adjustment or stratification was needed.

**Sleep measurements**: Sleep was assessed using parent-rated questionnaires and actigraphy at mean age of 11.1 years (range 9.8-12.7). We used parent-reported Sleep Disturbances Scale for Children (SDSC)^34^ to assess difficulties of initiating and maintaining sleep and sleep fragmentation. The SDSC consists of 26 items that quantify sleep problems in a 5-Likert scale over the past 6 months. Also, parents reported “How long does your child sleep at night” and we used this information to assess reported sleep duration.

Preadolescents wore a tri-axial wrist accelerometer (GENEActiv; Activinsights, UK) on their non-dominant wrist and completed a sleep diary for 7 consecutive days (Cabré-Riera et al. 2021, submitted). Measurements were processed using the R-package *GGIR*.^20^ We obtained objective sleep measures for each day which included total sleep time (time between falling asleep and final awakening from which the time spent awake in between is subtracted, in hours), sleep onset latency (time between lying down in bed and falling asleep, in minutes), and wake after sleep onset (time awake between falling asleep and final awakening, in minutes). We calculated the mean of each objective sleep measure across 7 days. Moreover, we collected information on sleep quality (“How did you sleep last night?”), categorized as very good, good, or regular/bad/very bad, and restfulness (“How rested do you feel this morning?”), categorized as very well rested, rested, or moderately/poorly/very poorly rested, for 7 consecutive days using a sleep diary completed by preadolescents.

**DNA sample collection:** Cord blood and whole blood collected at age 4y was extracted using the Chemagen kit (Perkin Elmer). DNA concentration was determined by a NanoDrop spectrophotometer (Thermo Scientific) and with the Quant-iT PicoGreen dsDNA Assay Kit (Life Technologies).

**Methylation measurements**: Blood methylation data was produced in two laboratories: the Genome Analysis Facility of the University Medical Center Groningen (UMCG) in the Netherlands as part of the MeDALL project (0y and 4y), and the Bellvitge Biomedical Research Institute (IDIBELL) in Barcelona as part of the BREATHE project (0y). Both laboratories randomized the samples in batches and followed the Illumina protocol for the Infinium HumanMethylation450 BeadChip. Briefly, 500 ng of DNA was bisulfite-converted using the EZ 96-DNA methylation kit, and DNA methylation was measured through hybridization on the BeadChips. BeadChips were scanned with an Illumina iScan and image data was uploaded into the Methylation Module of Illumina’s analysis software GenomeStudio, and converted in β-values. Two blood samples with overall low quality (*MethylAid* package),^29^ and three blood samples discordant for sex (*shinyMethyl* package)^31^ were removed. After applying a stringent detection p-value of 1.10E-16,^13^ 18 blood samples with a call rate <98% were excluded. 7,136 probes with a call rate <95%, control probes and probes designed to detect genetic polymorphisms were removed. Data was normalized with the functional normalization method with prior background correction with *Noob* implemented in the *minfi* package.^14^

Due to issues arising from the very small number of participants with DNAm data in childhood and caregiver-reported sleep initiation or fragmentation problems, these models were dropped during cohort-level quality control. Further, as the same individuals who form the INMA cohort were already included in the analyses on childhood DNAm and caregiver-reported sleep duration as part of the multi-center HELIX cohort, we did not analyze the associations of childhood DNAm with caregiver-reported child sleep in INMA.

**Cell type correction**: Estimation of blood cell type proportions was obtained from methylation data applying the Houseman method,^15^ and the Bakulski^17^ and Reinius^21^ reference panels for cord blood and child blood, respectively.

**Batch correction**: We used *ComBat* algorithm^32^ to adjust for potential batch effects, using laboratory as the major known technical bias.

**Ancestry/ethnicity**: No ancestry covariates were included in the models: all participants were of European ancestry.

**Maternal education**: Maternal education was assessed at child birth. We categorized maternal education on high (University level or higher), medium (secondary education), or low (primary education, lower or no education)

**Smoking during pregnancy**: Maternal self-reported smoking status during pregnancy was classified into never smoked during pregnancy vs smoked during pregnancy.

**Gestational age at birth**: Gestational age was calculated from the date of the last menstrual period (LMP) reported at recruitment and confirmed using estimates based on ultrasound examination in the 12th week of gestation. When the difference between the LMP reported at recruitment and estimated from the ultrasound was ≥ 7 days, then LMP was estimated using a quadratic regression formula defined by Westerway et al.^35^

**Study funding**: This study was funded by grants from Instituto de Salud Carlos III (Red INMA G03/176; CB06/02/0041; FIS-FEDER-PI03-1615PI041436; FIS-FEDER-PI06/0867, PI081151 incl. FEDER funds; FIS-PI11/00610,PI12/01890 incl. FEDER funds; CP13/00054 incl. FEDER funds; PI15/00118 incl. FEDER funds; CP16/00128 incl. FEDER funds; PI16/00118 incl. FEDER funds; PI16/00261 incl. FEDER funds; PI17/01340, PI18/00547 incl. FEDER funds, CPII18/00018), CIBERESP, Generalitat de Catalunya-CIRIT 1999SGR 00241, Generalitat de Catalunya-AGAUR (2009 SGR 501, 2014 SGR 822), Fundació La marató de TV3 (090430), Spanish Ministry of Economy and Competitiveness (SAF2012-32991 incl. FEDER funds), Agence Nationale de Securite Sanitaire de l’Alimentation de l’Environnement et du Travail (1262C0010; EST-2016 RF-21), EU Commission (261357, 308333, 603794, 634453, and 824989, 874583), and European Research Council (268479). ISGlobal acknowledges support from the Spanish Ministry of Science and Innovation through the “Centro de Excelencia Severo Ochoa 2019-2023” Program (CEX2018-000806-S), and support from the Generalitat de Catalunya through the CERCA Program.

**Acknowledgements**: INMA researchers would like to thank all the participants for their generous collaboration. A full roster of the INMA Project Investigators can be found at <http://www.proyectoinma.org/presentacioninma/listado-investigadores/en_listado-investigadores.html>

# LINA

**Full name:** Lifestyle and environmental factors and their Influence on Newborns Allergy risk

**Cohort profile**: Hinz et al (2012)^36^

**Design and study population**: The LINA study (**L**ifestyle and Environmental Factors and their **I**nfluence on **N**ewborn’s **A**llergy Risk) is a running prospective birth cohort study conducted by the Helmholtz Centre for Environmental Research-UFZ in Leipzig, in cooperation with clinical partners with the aim to entangle the impact of environmental factors on the maturation of children’s immune system and the development of allergic diseases, obesity and behavioral disorders. For this study, 629 mother-child pairs were recruited from March 2006 until December 2008 in the city of Leipzig, Germany. Pregnant women were invited to participate, and after informed consent, maternal blood and urine samples as well as questionnaire data were collected between the 34^th^-36^th^ weeks of gestation. Since birth, children as well as mothers are followed up annually by standardized questionnaires and clinical visits including blood, urine and feces collection. In maternal and in children’s blood, several immune parameters, endogenous metabolites, gene expression, Vitamin D and other parameters are measured in each follow up. The concentration of environmental chemicals like phthalates and parabens have been measured in maternal urine from pregnancy. Methylation analyses (450K analysis) have been performed in cord blood samples.

**Consent and ethical approval**: Written informed consent was obtained from all participating parents. The LINA Study is conducted in accordance with the World Medical Association Declaration of Helsinki and was approved by the Ethical Committee of the University of Leipzig.

**Division into subcohorts**: None

**Sleep measurements**: Information on sleep behavior was obtained from parent-rated questionnaires at the mean age of 10.1 years (range 9.7-11.5 years). We used information on mean sleep duration during the respective period distinguished by day or night time.

**DNA sample collection:** DNA was extracted from cord blood collected at birth.

**Methylation measurements**: We used the Illumina Infinium® HumanMethylation450 BeadChip to measure DNA methylation in cord blood.

**Cell type correction**: The algorithm described by Houseman et al. together with the cord blood DNAm reference data set provided by Bakulski et al. were used to estimate cell type proportions.

**Batch correction**: Adjustment for batch effects was done by surrogate variables for technical batch, using the *sva* package in R.^5^

**Ancestry/ethnicity**: No adjustment for ethnicity was made, because all LINA participants are of the same European/Caucasian ethnicity.

**Maternal education**: Maternal education was measured according to the ISCED recommendations and transformed to a 0-1 binary measure.

**Smoking during pregnancy**: Smoking during pregnancy was obtained from a self-report questionnaire.

**Gestational age at birth**: Gestational age at birth was obtained from medical records ("*Mutterpass*").

**Study funding**: The LINA study is funded by the Helmholtz Centre for Environmental Research - UFZ. Parts of the LINA study were funded by the Helmholtz Impulse and Networking Fund as well as by the German Research Foundation (KFO250). EWAS analyses in the LINA study were funded by the German Cancer Research Center - DKFZ.

**Acknowledgements**: We cordially thank the participants of the LINA study as well as their parents, the midwives and the physicians. We are grateful to Beate Fink, Anne Hain, Michaela Loschinski, Sandra Albrecht, Melanie Bänsch and André Andrecke for their excellent technical assistance and fieldwork.

# MoBa-1 and MoBa-2

**Full name:** Norwegian Mother, Father and Child Cohort Study

**Cohort profile**: Magnus et al (2016),^37^ Rønningen (2006)^38^

**Design and study population**: The prospective population-based Norwegian Mother, Father and Child Cohort Study (MoBa) (formerly Mother and Child Cohort Study) includes women who were initially recruited during a routine antenatal ultrasound examination, and their families. The recruitment commenced in a large hospital in the city of Bergen, Norway, in the summer of 1999 and gradually expanded to include 50 of Norway’s 52 hospitals with maternity units: the first participating child was born in October 1999 and the last in July 2009.

**Consent and ethical approval**: The establishment and data collection in MoBa has obtained a license from the Norwegian Data Inspectorate and approval from The Regional Committee for Medical Research Ethics in Norway, and written informed consent was provided by all mothers participating. The MoBa1 and MoBa2 subcohorts were also approved by the Institutional Review Board of the National Institute of Environmental Health Sciences, USA.

**Division into subcohorts**: For the current study, two subsets of the MoBa cohort were examined: the MoBa1 and MoBa2 study populations. MoBa1 is part of a larger study within MoBa that was designed to evaluate the association between maternal plasma folate during pregnancy and childhood asthma status at 3 years of age.^39^ MoBa2 included a cohort random sample plus cases of asthma at age seven years and non-asthmatic controls.^40^ The current analyses include the children who had cord blood DNA methylation measurements and covariate data, and each dataset was analyzed independently.

**Sleep measurements**: Sleep was assessed using parent-rated questionnaires at 5.0-8.0 years of age (mean age of 7.0 years in MoBa-1, and 7.1 years in MoBa-2). The questionnaire included one sleep-related item: "Approximately how many hours of sleep does the child usually sleep on a week night?", coded into 8 ("8 hours or less"), 9, 10, 11, or 12 ("12 hours or more") and standardized within the cohort (mean=0, SD=1).

**DNA sample collection:** DNA was extracted from cord blood collected at birth.

**Methylation measurements**: We used the *Illumina Infinium® HumanMethylation450 BeadChip* to measure DNA methylation in cord blood. Details of the DNA methylation measurements and quality control for the MoBa1 participants were previously described^41^ and the same protocol was implemented for the MoBa2 participants. Briefly, umbilical cord blood samples were collected and frozen at birth at -80°C in line with the procedures at the Biobank of MoBa.^42^ Bisulfite conversion was performed using the EZ-96 DNA Methylation kit (Zymo Research Corporation, Irvine, CA) and DNA methylation was measured at 485,577 CpGs in cord blood using the Illumina Infinium® HumanMethylation450 BeadChip. Raw intensity (.*idat*) files were handled in R using the *minfi* package^14^ to calculate the methylation level at each CpG as the beta-value (β=intensity of the methylated allele (M)/(intensity of the unmethylated allele (U) + intensity of the methylated allele (M) + 100)) and the data was exported for quality control and processing.

Probe and sample-specific quality control was performed in the MoBa1 and MoBa2 datasets separately. Similar protocols were applied to MoBa1 and Moba2, as follows: Control probes (N=65) and probes on X (N=11,230) and Y (N=416) chromosomes were excluded in both datasets. Remaining CpGs missing > 10% of methylation data were also removed (N=20 in MoBa1, none in MoBa2). Samples indicated by Illumina to have failed or have an average detection p value across all probes < 0.05 (N=49 MoBa1, N=35 MoBa2) and samples with sex mismatch (N=13 MoBa1, N=8 MoBa2) were also removed. For MoBa1 and MoBa2, we accounted for the two different probe designs by applying the intra-array normalization strategy Beta Mixture Quantile dilation (BMIQ).^43^

**Cell type correction**: Seven cell types (B, CD4+ T, CD8+ T, granulocytes, monocytes, NK and nucleated red blood cells- nRBC) proportions were determined using the Houseman method^15^ with the Bakulski cord blood reference panel^17^ through the *minfi* package.^14^

**Batch correction**: Adjustment for batch effects was done using *ComBat*^32^ and surrogate variable analysis using the *sva* package in R.^5^

**Ancestry/ethnicity**: Due to the ethnic homogeneity of the cohort, ethnicity was not included in the analyses.

**Maternal education**: Based on self-reported education level during pregnancy, maternal education was categorized into low (high school or lower) vs medium/high (college or higher). Due to the low number of mothers with primary education only, categorization into ISCED level 0-2 vs level 3-8 was not feasible.

**Smoking during pregnancy**: Mothers' self-reported smoking during pregnancy was categorized into no smoking during pregnancy, vs quit in early pregnancy, vs continued after early pregnancy.

**Gestational age at birth**: Gestational age was calculated using ultrasound estimation. If ultrasound was not available, last menstrual period was used.

**Study funding**: The Norwegian Mother, Father and Child Cohort Study are supported by the Norwegian Ministry of Health and Care Services and the Ministry of Education and Research, NIH/NIEHS (contract no N01-ES-75558), NIH/NINDS (grant no.1 UO1 NS 047537-01 and grant no.2 UO1 NS 047537-06A1). For this work, MoBa 1 and 2 were supported by the Intramural Research Program of the NIH, National Institute of Environmental Health Sciences (Z01-ES-49019) and the Norwegian Research Council/BIOBANK (grant no 221097). The work was partly funded by The Norwegian Research council’s Centre of Excellence Scheme (grant no 262700). The funders had no role in the planning or execution of the study nor the interpretation or publication of its results.

**Acknowledgements**: We are grateful to all the participating families in Norway who take part in this on-going cohort study.

# PREDO

**Full name:** Prediction and Prevention of Preeclampsia and Intrauterine Growth Restriction

**Cohort profile**: Girchenko et al (2017)^44^

**Design and study population**: The Prediction and Prevention of Preeclampsia and Intrauterine Growth Restriction (PREDO) study is a prospective, multicenter study of Finnish women who were pregnant between 2005 and 2009 and their children. PREDO recruited 1079 women with a singleton, intrauterine pregnancy, who visited antenatal clinics at any of the 10 study hospitals for their first routine ultrasound screening at 12 to 13 weeks of gestation, of whom 969 had one or more and 110 had none of the known risk factors for preeclampsia and intrauterine growth restriction.^44^ The children who were younger than 3 years old at time of caregiver-reported sleep were excluded from the analytic sample.

**Consent and ethical approval**: All participating mothers provided written informed consent. The study protocol was approved by the Ethics Committee of Obstetrics and Gynaecology and Women, Children and Psychiatry of the Helsinki and Uusimaa Hospital District and by the participating hospitals. The study has been registered as ClinicalTrials.gov identifier ISRCTN14030412.

**Division into subcohorts**: No. The original PREDO cohort is divided into two subsamples: subsample with known risk factor status for pre-eclampsia and community-based subsample. All the participants in the analytic sample for the current study, however, belong to the subsample with known risk factor status for pre-eclampsia.

**Sleep measurements**: Sleep was assessed using parent-rated questionnaires at mean age of 3.8 years (range 3.0-5.8). Participants reported how long the child sleeps at night, in hours and minutes. The sleep duration was standardized and winsorized within the sample. Parents completed the Sleep Disturbance Scale for Children (SDSC).^34^ SDSC item 4 “The child has difficulty getting to sleep at night” was dichotomized into no (never/occasionally) vs yes (sometimes/often/always) and used for sleep initiation difficulties outcome. SDSC item 10 “The child wakes up more than twice per night” was used for sleep fragmentation outcome: this item was dichotomized as no (never) vs yes (occasionally/sometimes/often/always): this dichotomization was chosen as only a very small number of participants selected ‘sometimes’, ‘often’ or ‘always’.

**DNA sample collection:** DNA was extracted from cord blood collected at birth.

**Methylation measurements**: We used the *Illumina Infinium® HumanMethylation450 BeadChip* to measure DNA methylation in cord blood. To limit batch effects, we randomized all samples over the 96-well plates, based on gender and maternal risk factors for pre-eclampsia. Samples were placed on 96-well plates. Bisulfite conversion was performed using the EZ-96 DNA methylation kit (Zymo research Corporation, Irvine, USA). Then we used the *Infinium HumanMethylation450 BeadChip* (Illumina Inc., San Diego, USA) to measure the methylation level as a beta value ranging from 0 (no methylation) to 1 (complete methylation). The quality control pipeline was set up using the R-package *minfi.*^14^ Three IDs were excluded as they were outliers in the median intensities. Furthermore, 20 IDs showed disconcordance between phenotypic sex and estimated sex and were excluded. Nine IDs were contaminated with maternal DNA and were also removed.^45^ Methylation beta-values were normalized using the *funnorm* function. We excluded any probes on chromosome X or Y, probes containing SNPs and cross-hybridizing probes according to Chen et al.^24^ and Price et al.^25^ Furthermore, any CpGs with a detection p-value > 0.01 in at least 25% of the samples were excluded. After normalization two batches, i.e., slide and well, were significantly associated and were removed iteratively using the *ComBat* method.

**Cell type correction**: The Houseman method with the Bakulski cord blood reference panel^17^ was used to estimate the cell composition in the cord blood.

**Batch correction**: The *ComBat* method in the *sva* package in R was used for the batch correction.

**Ancestry/ethnicity**: Three first principal components from genotyped data were used as ancestry covariates.

**Maternal education**: Maternal socioeconomic class based on self-reported level of education during pregnancy, classified into primary, secondary, lower tertiary, or upper tertiary: this was then dichotomized into low (ISCED level 0-2) vs medium/high (ISCED level 3-8).

**Smoking during pregnancy**: Maternal smoking status was based on data recorded in the Finnish Medical Birth Register and classified into none, vs quit during the first trimester, vs continued smoking after the first trimester.

**Gestational age at birth**: The data on gestational age at birth came from the Finnish Medical Birth Register.

**Study funding**: The PREDO study has received funding from the Academy of Finland, EraNet, EVO (a special state subsidy for health science research), University of Helsinki Research Funds, the Signe and Ane Gyllenberg foundation, the Emil Aaltonen Foundation, the Finnish Medical Foundation, the Jane and Aatos Erkko Foundation, the Novo Nordisk Foundation, the Päivikki and Sakari Sohlberg Foundation, the Sigrid Juselius Foundation, and the Sir Jules Thorn Charitable Trust.

**Acknowledgements**: We thank all the children and their parents for participation. We also thank all the research nurses, research assistants, and laboratory personnel involved in the PREDO study.

# PROGRESS

**Full name:** Programming Research in Obesity, Growth, Environment and Social Stressors study

**Cohort profile**: Rosa-Parra et al (2018),^46^ Tamayo y Ortiz et al (2017)^47^

**Design and study population**: *Programming Research in Obesity, GRowth Environment and Social Stress* (PROGRESS) is a prospective birth cohort of healthy pregnant women. We recruited women if they were less than 20 weeks of gestation through the Mexican Social Security System between July 2007 and February 2011. We enrolled women older than 18 years who have access to a telephone and a plan to reside within Mexico City for the following 3 years. We excluded people with diagnosis of heart or kidney disease, use of steroids or anti-epilepsy drugs, or daily alcohol consumption.

**Consent and ethical approval**: Study protocols were approved by the institutional review boards of the Icahn School of Medicine at Mount Sinai, Harvard T. H. Chan School of Public Health, the National Institute of Public Health in Mexico, the Mexican Social Security System, and the National Institute of Perinatology in Mexico. All participating mothers provided written informed consent.

**Division into subcohorts**: None.

**Sleep measurements**: Sleep was assessed using actigraphy at mean age of 4.7 years (range 4.0-6.7 years). Sleep variables were measured using Actigraph GT3X (ActiGraph, Inc, Pensacola, FL) wrist-worn accelerometers. The ActiGraph measured motion in oscillations as well as ambient light. Trained physicians delivered the accelerometers in-person to each participant, and placed the devices on the non-dominant wrist using Hospital-style wrist straps to prevent children from removing the ActiGraph device during the full wear-period. Additionally, mothers were instructed to supervise that their children wore the accelerometer 24 hours a day for 7 days without removing it at any time, and to record any sleep time using a sleep diary. The parents were given additional bracelets in case the accelerometer had to be taken off and put back on during the observation period (e.g., the child reported that the bracelet was too tight). Research staff retrieved the accelerometer during a home visit after the full wear period was over (7 full days, starting the day after delivery).

To calculate an estimate of sleep duration, we identified sustained periods with low to no physical activity (defined as <1000 oscillations per minute). Using accelerometry software ActiLife v6.11.9, we used the Sadeh sleep algorithm to estimate the number of minutes the child slept within each sleep period. The “time in bed” variable was produced by manually scoring the accelerometer data based on a standardized protocol we developed with input from the sleep laboratory at Brigham and Women’s Hospital. We used reductions and increases in the number of counts of physical activity measured in each 60-second epoch to bookend when the child lay down at night and got up in the morning. We used the first 60-second epoch with <1000 counts of physical activity as the start of a “time in bed” period and the last epoch with <1000 counts as the end of that period. We then calculated child sleep efficiency (% of in bed time spent asleep) by dividing time asleep (as determined by accelerometry) by the total time in bed. This approach with the Actigraph GT3x has been validated previously against polysomnography to assess sleep duration and efficiency in adults. Nap times were defined as sleep time less than 360 minutes/6 hours and were removed to avoid duplicate observation days that can result from nap times.

Total Sleep Time, Sleep Onset Latency, and Wake After Sleep Onset were measured in minutes. Total Sleep Time was transformed into hours. We calculated average Total Sleep Time, Sleep Onset Latency, and Wake After Sleep Onset across the observation period. The target duration of the observation period was seven nights, however we allowed missing data for a maximum of one night, i.e., anyone with usable data for 6-7 nights was included in the analyses (n=244, or 87% of the 279 children who participated in the assessment). Total Sleep Time, Sleep Onset Latency, and Wake After Sleep Onset variables were z-standardized using the formula of subtracting the mean from the score and dividing by standard deviation.

**DNA sample collection:** DNA was extracted from cord blood collected at birth.

**Methylation measurements**: We used the *Illumina Infinium® HumanMethylationEPIC BeadChip* to measure DNA methylation in cord blood. Quality control included the following steps: 1) Exclusion of failed samples using Illumina control probes monitoring experimental steps, 2) Identification and exclusion of mislabeled and contaminated samples,^48^ including sex mismatches, and 3) Removal of outliers in PCA plot. We filtered observations with detection p-values above 0.01 using non-specific fluorescence to estimate background noise.^49^ Dye-bias correction was done using the *RELIC* method.^50^ After these steps, no normalization was deemed necessary.

**Cell type correction**: We used the Houseman algorithm to estimate cell composition.^15^ In the cord blood DNAm analyses, we used a combined reference panel, using both the Bakulski cord blood reference panel^17^ and the de Goede cord blood reference panel^51^ to estimate cord blood cell composition.

**Batch correction**: The first 20 surrogate variables were included as covariates.

**Ancestry/ethnicity**: The cohort is of Mexican ethnicity. No ancestry covariates were included in the model.

**Maternal education**: Maternal education was based on self-reports from the General questionnaire; mothers were asked about their highest level of educational attainment and the highest grade they reached for each education level. Based on this information, we categorized a variable as follows: (1) less than high school, (2) high school, (3) more than high school.

**Smoking during pregnancy**: Due to the low number of smokers among the recruited women, no such variable was included in the model.

**Gestational age at birth**: We used self-report of the last menstrual period to determine gestational age. For a subset, however, we used the Capurro method to compensate for recall bias regarding last menstrual period. If the last menstrual period and Capurro method differed by more than 3 weeks, the Capurro method was used.

**Study funding**: National Institute of Environmental Health Sciences (P30ES023515, R01ES020268, R01ES013744, R01ES014930, R01ES021357, R24ES028522; R00ES023450).

**Acknowledgements**: We acknowledge the American British Cowdray Medical Center for providing research facilities, which made it possible to conduct the study.

# Project Viva

**Full name:** Project Viva

**Cohort profile**: Oken et al (2015)^52^

**Design and study population**: Project Viva is a longitudinal pre-birth cohort established to examine the effects of events during early development on lifetime health outcomes. Between April 1999 and November 2002, the study recruited women in early pregnancy from eight obstetric offices of Atrius Harvard Vanguard Medical Associates, a multispecialty group practice in eastern Massachusetts. Exclusion criteria included multiple gestation, inability to answer questions in English, gestational age ≥22 weeks at recruitment, and plans to move away from the study area before delivery. Of 2670 enrolled participants, 2128 were still enrolled at delivery and had a live birth.

**Consent and ethical approval**: All mothers provided written informed consent and children provided verbal assent for the mid-childhood blood draw. Institutional review boards at all participating institutions gave approval for this study.

**Division into subcohorts**: None

**Sleep measurements**: Sleep was assessed using parent-rated questionnaires at mean age of 7.8 years (range 6.7 to 10.6 years). Parents reported the number of hours their child slept in a usual 24-hour period on an average weekday and weekend day in the past month. We calculated sleep hours/day as (weekday hours/day*5 + weekend hours/day*2)/7.^53^ For the analysis, sleep duration was z-standardized and winsorized. Four sleep values were winsorized in cord blood analysis, and 3 sleep values were winsorized in child whole blood analysis.

**DNA sample collection:** DNA was extracted from cord blood collected at birth, and from child blood samples collected through venipuncture in mid-childhood (mean age of 7.8 years, range 6.7 to 10.6 years). At birth, cord blood was collected by obstetricians and midwives from the umbilical cord vein and centrifuged within 24 hours of collection. Samples included in this analysis had a mean gestational age of 39.7 weeks (range 30.9 to 42.6). At the mid-childhood visit, trained research assistants obtained whole blood from the antecubital vein, and included participants had a mean age of 7.8 (range 6.7 to 10.6) at the time of the blood draw. Genomic DNA was extracted from nucleated cells using commercially available Qiagen PureGene Kits (Valencia, CA) and frozen at -80 degrees C. DNA underwent sodium bisulfite conversion using the EZ DNA Methylation-Gold Kit (Zymo Research, Irvine, CA).

**Methylation measurements**: We used the *Illumina Infinium® HumanMethylation450 BeadChip* to measure DNA methylation in cord blood and child blood. Data were processed using the *minfi* package in R. We performed background and dye-bias correction via normal-exponential out-of-band processing (*noob*) and corrected for probe-type bias using the b-mixture quantile intra sample normalization procedure (*BMIQ*) method. We excluded samples that failed on the array (cord blood n= 4, mid-childhood blood n=5), that were low-quality (cord blood n=10, mid-childhood blood n=6), that had a genotype mismatch when compared with a blood sample from the same individual at a different timepoint (cord blood n=6, mid-childhood blood n=4) or that had a predicted sex mismatch with recorded sex (cord blood n=6, mid-childhood n=3). Overall, Project Viva contributed cord blood DNA methylation from 410 newborns, and childhood blood DNA methylation from 438 children to the current PACE meta-analyses on child sleep. For probes quality control, we excluded probes that were on the sex chromosomes or that had a detection p-value less than 0.05 in greater than 5% of samples, contributing a total of 467,471 probes to the meta-analysis.

**Cell type correction**: We corrected for seven types of cells in cord blood using the Bakulski cord blood reference panel, and for six types of cells in the mid-childhood blood using the Houseman blood reference panel.

**Batch correction**: We used *ComBat* to adjust for sample plate while protecting the sleep duration variable, as well as sex of proband.

**Ancestry/ethnicity**: Project Viva represents a mixed-ancestry population. Based on parent report, in the cord blood and childhood analytical samples, respectively, 68.7% and 61.6% of children were White, 12.7% and 19.4% were Black, 3.7% and 3.2% were Asian, 4.6% and 5.3% were Hispanic, and 10.3% and 10.5% had a multiracial background. Parent-reported child race/ethnicity was included as a covariate in the analyses.

**Maternal education**: Mothers reported their highest education level based on a 5-point ordinal scale (1 = Less than 12th grade, 2 = High school degree or a GED, 3 = Some college, 4 = 4 years of college, 5 = Graduate degree). Based on the analytic plan, we dichotomized education as 1 = at least a high school degree (categories 2-5) vs. 0 = less than a high school degree (category 1).

**Smoking during pregnancy**: Maternal self-reported smoking status during pregnancy was classified into ‘never smoked during pregnancy’ vs. ‘ever smoked during pregnancy’. We were unable to treat ‘continued smoking’ and ‘quit smoking during pregnancy’ as separate categories due to the extremely small number of mothers who reported continued smoking in Project Viva.

**Gestational age at birth**: We obtained child date of birth from the hospital medical records. We calculated gestational age by using the date of the last menstrual period, but if the early second-trimester ultrasound assessment differed from the calculated gestational age by more than 10 days, we used the ultrasound dating instead.

**Study funding**: Grants from the US National Institutes of Health (R01 HD034568, UH3 OD023286, R01 HL111108, R01 NR013945).

**Acknowledgements**: We thank the participants and staff of Project Viva.

# References

1 Boyd A, Golding J, Macleod J, Lawlor DA, Fraser A, Henderson J, et al. Cohort profile: The ’Children of the 90s’-The index offspring of the Avon longitudinal study of parents and children. *International Journal of Epidemiology* 2013;42:111–127.

2 Fraser A, Macdonald-Wallis C, Tilling K, Boyd A, Golding J, Davey smith G, et al. Cohort profile: The Avon longitudinal study of parents and children: ALSPAC mothers cohort. *International Journal of Epidemiology* 2013;42:97–110.

3 Relton CL, Gaunt T, McArdle W, Ho K, Duggirala A, Shihab H, et al. Data resource profile: Accessible resource for integrated epigenomic studies (ARIES). *International Journal of Epidemiology* 2015;44:1181–1190.

4 Min JL, Hemani G, Davey Smith G, Relton C, Suderman M. Meffil: Efficient normalization and analysis of very large DNA methylation datasets. *Bioinformatics* 2018;34:3983–3989.

5 Leek JT, Johnson WE, Parker HS, Jaffe AE, Storey JD. The SVA package for removing batch effects and other unwanted variation in high-throughput experiments. *Bioinformatics* 2012;28:882–883.

6 Koletzko B, Kries V, Closa R, Escribano J, Scaglioni S, Giovannini M, et al. Lower protein in infant formula is associated with lower weight up to age 2 y: a randomized clinical trial. *American Journal of Clinical Nutrition* 2009;89:1836–1845.

7 Weber M, Grote V, Closa-Monasterolo R, Escribano J, Langhendries JP, Dain E, et al. Lower protein content in infant formula reduces BMI and obesity risk at school age: follow-up of a randomized trial. *American Journal of Clinical Nutrition* 2014;99:1041–51.

8 Rzehak P, Saffery R, Reischl E, Covic M, Wahl S, Grote V, et al. Maternal smoking during pregnancy and DNA-methylation in children at age 5.5 years: Epigenome-wide-analysis in the European Childhood Obesity Project (CHOP)-study. *PLoS ONE* 2016;11:1–18.

9 Graef DM, Janicke DM, McCrae CS. Sleep patterns of a primarily obese sample of treatment-seeking children. *Journal of Clinical Sleep Medicine* 2014;10:1111–1117.

10 Soric M, Turkalj M, Kucic D, Marusic I, Plavec D, Misigoj-Durakovic M. Validation of a multi-sensor activity monitor for assessing sleep in children and adolescents. *Sleep Medicine* 2013;14:201–205.

11 Roane BM, Van Reen E, Hart CN, Wing R, Carskadon MA. Estimating sleep from multisensory armband measurements: Validity and reliability in teens. *Journal of Sleep Research* 2015;24:714–721.

12 Meltzer LJ, Montgomery-Downs HE, Insana SP, Walsh CM. Use of actigraphy for assessment in pediatric sleep research. *Sleep Medicine Reviews* 2012;16:463–475.

13 Lehne B, Drong AW, Loh M, Zhang W, Scott WR, Tan ST, et al. A coherent approach for analysis of the Illumina HumanMethylation450 BeadChip improves data quality and performance in epigenome-wide association studies. *Genome Biology* 2015;16:1–12.

14 Aryee MJ, Jaffe AE, Corrada-Bravo H, Ladd-Acosta C, Feinberg AP, Hansen KD, et al. Minfi: A flexible and comprehensive Bioconductor package for the analysis of Infinium DNA methylation microarrays. *Bioinformatics* 2014;30:1363–1369.

15 Houseman EA, Accomando WP, Koestler DC, Christensen BC, Marsit CJ, Nelson HH, et al. DNA methylation arrays as surrogate measures of cell mixture distribution. *BMC Bioinformatics* 2012;13.

16 Heude B, Forhan A, Slama R, Douhaud L, Bedel S, Saurel-Cubizolles MJ, et al. Cohort Profile: The EDEN mother-child cohort on the prenatal and early postnatal determinants of child health and development. *International Journal of Epidemiology* 2016;45:353–363.

17 Bakulski KM, Feinberg JI, Andrews S V., Yang J, Brown S, L. McKenney S, et al. DNA methylation of cord blood cell types: Applications for mixed cell birth studies. *Epigenetics* 2016;11:354–362.

18 Kooijman MN, Kruithof CJ, van Duijn CM, Duijts L, Franco OH, van IJzendoorn MH, et al. The Generation R Study: Design and cohort update 2017. *European Journal of Epidemiology* 2016;31:1243–1264.

19 Koopman-Verhoeff ME, Mulder RH, Saletin JM, Reiss I, van der Horst GTJ, Felix JF, et al. Genome-wide DNA methylation patterns associated with sleep and mental health in children: a population-based study. *Journal of Child Psychology and Psychiatry and Allied Disciplines* 2020;61:1061–1069.

20 Van Hees VT, Fang Z, Langford J, Assah F, Mohammad A, Da Silva ICM, et al. Autocalibration of accelerometer data for free-living physical activity assessment using local gravity and temperature: An evaluation on four continents. *Journal of Applied Physiology* 2014;117:738–744.

21 Reinius LE, Acevedo N, Joerink M, Pershagen G, Dahlén SE, Greco D, et al. Differential DNA methylation in purified human blood cells: Implications for cell lineage and studies on disease susceptibility. *PLoS ONE* 2012;7.

22 Strandberg TE, Järvenpää AL, Vanhanen H, McKeigue PM. Birth outcome in relation to licorice consumption during pregnancy. *American Journal of Epidemiology* 2001;153:1085–1088.

23 Pesonen AK, Martikainen S, Heinonen K, Wehkalampi K, Lahti J, Kajantie E, et al. Continuity and change in poor sleep from childhood to early adolescence. *Sleep* 2014;37:289–297.

24 Chen YA, Lemire M, Choufani S, Butcher DT, Grafodatskaya D, Zanke BW, et al. Discovery of cross-reactive probes and polymorphic CpGs in the Illumina Infinium HumanMethylation450 microarray. *Epigenetics* 2013;8:203–209.

25 Price ME, Cotton AM, Lam LL, Farré P, Emberly E, Brown CJ, et al. Additional annotation enhances potential for biologically-relevant analysis of the Illumina Infinium HumanMethylation450 BeadChip array. *Epigenetics and Chromatin* 2013;6:1–15.

26 McCartney DL, Walker RM, Morris SW, McIntosh AM, Porteous DJ, Evans KL. Identification of polymorphic and off-target probe binding sites on the Illumina Infinium MethylationEPIC BeadChip. *Genomics Data* 2016;9:22–24.

27 Maitre L, De Bont J, Casas M, Robinson O, Aasvang GM, Agier L, et al. Human Early Life Exposome (HELIX) study: A European population-based exposome cohort. *BMJ Open* 2018;8:1–17.

28 Vrijheid M, Slama R, Robinson O, Chatzi L, Coen M, Van Den Hazel P, et al. The Human Early-Life Exposome (HELIX): Project Rationale and Design. *Environmental Health Perspectives* 2014;122:535–544.

29 Van Iterson M, Tobi EW, Slieker RC, Den Hollander W, Luijk R, Slagboom PE, et al. MethylAid: Visual and interactive quality control of large Illumina 450k datasets. *Bioinformatics* 2014;30:3435–3437.

30 Triche TJ, Weisenberger DJ, Van Den Berg D, Laird PW, Siegmund KD. Low-level processing of Illumina Infinium DNA Methylation BeadArrays. *Nucleic Acids Research* 2013;41:1–11.

31 Fortin J-P, Fertig E, Hansen K. shinyMethyl: interactive quality control of Illumina 450k DNA methylation arrays in R. *F1000Research* 2014;3:175.

32 Johnson WE, Li C, Rabinovic A. Adjusting batch effects in microarray expression data using empirical Bayes methods. *Biostatistics* 2007;8:118–127.

33 Guxens M, Ballester F, Espada M, Fernández MF, Grimalt JO, Ibarluzea J, et al. Cohort profile: The INMA-INfancia y Medio Ambiente-(environment and childhood) project. *International Journal of Epidemiology* 2012;41:930–940.

34 Bruni O, Ottaviano S, Guidetti V, Romoli M, Innocenzi M, Cortesi F, et al. The Sleep Disturbance Scale for Children (SDSC) construction and validation of an instrument to evaluate sleep disturbances in childhood and adolescence. *Journal of Sleep Research* 1996;5:251–261.

35 Westerway SC, Davison A, Cowell S. Ultrasonic fetal measurements: New Australian standards for the new millennium. *Australian and New Zealand Journal of Obstetrics and Gynaecology* 2000;40:297–302.

36 Hinz D, Bauer M, Röder S, Olek S, Huehn J, Sack U, et al. Cord blood Tregs with stable FOXP3 expression are influenced by prenatal environment and associated with atopic dermatitis at the age of one year. *Allergy* 2012;67:380–389.

37 Magnus P, Birke C, Vejrup K, Haugan A, Alsaker E, Daltveit AK, et al. Cohort profile update: The Norwegian Mother and Child Cohort Study (MoBa). *International Journal of Epidemiology* 2016;45:382–388.

38 Rønningen KS, Paltiel L, Meltzer HM, Nordhagen R, Lie KK, Hovengen R, et al. The biobank of the Norwegian Mother and Child Cohort Study: A resource for the next 100 years. *European Journal of Epidemiology* 2006;21:619–625.

39 Håberg SE, London SJ, Nafstad P, Nilsen RM, Ueland PM, Vollset SE, et al. Maternal folate levels in pregnancy and asthma in children at age 3 years. *Journal of Allergy and Clinical Immunology* 2011;127:262–264.

40 Reese SE, Xu CJ, den Dekker HT, Lee MK, Sikdar S, Ruiz-Arenas C, et al. Epigenome-wide meta-analysis of DNA methylation and childhood asthma. *Journal of Allergy and Clinical Immunology* 2019;143:2062–2074.

41 Joubert BR, Håberg SE, Nilsen RM, Wang X, Vollset SE, Murphy SK, et al. 450K epigenome-wide scan identifies differential DNA methylation in newborns related to maternal smoking during pregnancy. *Environmental Health Perspectives* 2012;120:1425–1431.

42 Paltiel L, Haugan A, Skjerden T, Harbak K, Baekken S, Stensrud NK, et al. The biobank of the Norwegian Mother and Child Cohort Study – present status. *Norsk Epidemiologi* 2014;24:29–35.

43 Teschendorff AE, Marabita F, Lechner M, Bartlett T, Tegner J, Gomez-Cabrero D, et al. A beta-mixture quantile normalization method for correcting probe design bias in Illumina Infinium 450 k DNA methylation data. *Bioinformatics* 2013;29:189–196.

44 Girchenko P, Hämäläinen E, Kajantie E, Pesonen A-K, Villa PM, Laivuori H, et al. Prediction and Prevention of Preeclampsia and Intrauterine Growth Restriction (PREDO) study. *International Journal of Epidemiology* 2017;45:1380–1381.

45 Morin AM, Gatev E, McEwen LM, Macisaac JL, Lin DTS, Koen N, et al. Maternal blood contamination of collected cord blood can be identified using DNA methylation at three CpGs. *Clinical Epigenetics* 2017;9:1–9.

46 Rosa-Parra JA, Tamayo-Ortiz M, Lamadrid-Figueroa H, Cantoral-Preciado A, Montoya A, Wright RJ, et al. Diurnal cortisol concentrations and growth indexes of 12- to 48-month-old children from Mexico City. *Journal of Clinical Endocrinology and Metabolism* 2018;103:3386–3393.

47 Tamayo y Ortiz M, Téllez-Rojo MM, Trejo-Valdivia B, Schnaas L, Osorio-Valencia E, Coull B, et al. Maternal stress modifies the effect of exposure to lead during pregnancy and 24-month old children’s neurodevelopment. *Environment International* 2017;98:191–197.

48 Heiss JA, Just AC. Identifying mislabeled and contaminated DNA methylation microarray data: An extended quality control toolset with examples from GEO. *Clinical Epigenetics* 2018;10:1–9.

49 Heiss JA, Just AC. Improved filtering of DNA methylation microarray data by detection p values and its impact on downstream analyses. *Clinical Epigenetics* 2019;11:1–8.

50 Xu Z, Langie SAS, De Boever P, Taylor JA, Niu L. RELIC: A novel dye-bias correction method for Illumina Methylation BeadChip. *BMC Genomics* 2017;18:1–7.

51 de Goede OM, Razzaghian HR, Price EM, Jones MJ, Kobor MS, Robinson WP, et al. Nucleated red blood cells impact DNA methylation and expression analyses of cord blood hematopoietic cells. *Clinical Epigenetics* 2015;7:1–11.

52 Oken E, Baccarelli AA, Gold DR, Kleinman KP, Litonjua AA, Meo D De, et al. Cohort profile: Project Viva. *International Journal of Epidemiology* 2015;44:37–48.

53 Taveras EM, Gillman MW, Peña MM, Redline S, Rifas-Shiman SL. Chronic sleep curtailment and adiposity. *Pediatrics* 2014;133:1013–1022.
